# Genomic Analyses Uncover Evolutionary Features of Influenza A/H3N2 Viruses in Yunnan Province, China, from 2017 to 2022

**DOI:** 10.3390/v16010138

**Published:** 2024-01-18

**Authors:** Meiling Zhang, Jienan Zhou, Ruize Ni, Xiaonan Zhao, Yaoyao Chen, Yanhong Sun, Zhaosheng Liu, Xiaoyu Han, Chunrui Luo, Xiaoqing Fu, Yong Shao

**Affiliations:** 1 Department of Acute Infectious Diseases Control and Prevention, Yunnan Center for Disease Control and Prevention, Kunming 650022, China; meilingz2011@163.com (M.Z.); zhoujienanjj@163.com (J.Z.); nrzwangyi@163.com (R.N.); zhao_xiaonan@hotmail.com (X.Z.); chenyaoyao20231225@163.com (Y.C.); sunyanhong_lyy1225@163.com (Y.S.); lzslqyt@163.com (Z.L.); xiaoyuhxyy@163.com (X.H.); luochunrui123@163.com (C.L.); 2 State Key Laboratory of Genetic Resources and Evolution, Chinese Academy of Sciences, Kunming Institute of Zoology, Kunming 650201, China

**Keywords:** influenza A/H3N2 viruses, whole-genome sequencing analyses, genetic diversity, mutation, d_N_/d_S_, vaccine efficacy

## Abstract

Influenza A viruses evolve at a high rate of nucleotide substitution, thereby requiring continuous monitoring to determine the efficacy of vaccines and antiviral drugs. In the current study, we performed whole-genome sequencing analyses of 253 influenza A/H3N2 strains from Yunnan Province, China, during 2017–2022. The hemagglutinin (HA) segments of Yunnan A/H3N2 strains isolated during 2017–2018 harbored a high genetic diversity due to heterogeneous distribution across branches. The mutation regularity of the predominant antigenic epitopes of HA segments in Yunnan was inconsistent in different years. Some important functional mutations in gene segments associated with viral adaptation and drug tolerance were revealed. The rapid genomic evolution of Yunnan A/H3N2 strains from 2017 to 2022 mainly concentrated on segments, i.e., matrix protein 2 (M2), non-structural protein 1 (NS1), neuraminidase (NA), NS2, and HA, with a high overall non-synonymous/synonymous substitution ratio (d_N_/d_S_). Our results highlighted a decline in vaccine efficacy against the A/H3N2 circulating strains, particularly against the Yunnan 2021–2022 A/H3N2 strains. These findings aid our understanding of evolutionary characteristics and epidemiological monitoring of the A/H3N2 viruses and provide in-depth insights into the protective efficacy of influenza vaccines.

## 1. Introduction

According to the World Health Organization (WHO), 600 million influenza cases occur worldwide each year. Among these, 3 million suffer from severe illnesses, and the mortality is 290,000 to 650,000, with influenza A viruses being the most severe [1]. In China, the annual flu-related deaths in northern and southern cities stand at 18.0 and 11.3 per 100,000, respectively [2]. The influenza A viruses, which are characterized by a high genetic diversity, can be further classified into different subtypes based on two surface proteins, namely HA and NA [2]. Nowadays, 18 HA-based and 11 NA-based subtypes have been identified [3]. Studies have highlighted the predominance of the A/H3N2 subtype [4] as a significant contributor to seasonal influenza infections [5,6].

Influenza A viruses consist of multiple negative-strand RNA genomes. Their genomes comprise eight gene segments: HA, NA, M, nucleoprotein (NP), NS, polymerase basic 1 (PB1), polymerase basic 2 (PB2), and polymerase acidic (PA), which encode viral, RNA-dependent RNA polymerase and other proteins implicated in inducing cell apoptosis (e.g., PB1–F2) and regulating viral pathogenicity (e.g., PA-X) [7]. Among these encoded proteins, the HA harbors well-defined antigenic and receptor-binding sites, while the NA plays an important role in virus transmission via cleavage of the viral sialic acid receptor on the host cell membrane to facilitate the viral particles’ release [8,9]. The HA of influenza A/H3N2 virus possesses five antigenic epitopes (A-E), which are composed of the most variable amino acids subjected to host-neutralizing antibody-mediated immune pressure [10]. The continuous accumulation of mutations at these antigenic sites is mostly thought to drive antigenic drift among influenza viruses [11].

The annual administration of the multivalent influenza virus vaccine can mitigate the risk of morbidity and mortality, but its efficacy is contingent upon the degree of congruence between the strains selected for inclusion in the vaccine and those in circulation [12]. The rapid mutation rates of these viruses necessitate annual vaccine re-formulation, limiting its effectiveness to approximately 50% to 60% [13]. Various methodologies exist for estimating the antigenic distance between the strains used in influenza vaccines and those circulating in the population [14,15,16]. The P_epitope_ model, which quantifies amino acid changes in dominant epitopes between the vaccine and circulating strains, is considered more effective than phylogenetic analyses and antisera hemagglutination inhibition assays [12,17].

Yunnan Province, situated in southwestern China, shares a 4060 km land border with Myanmar, Laos, and Vietnam. This complex geography may contribute to regional diversity and divergence of the influenza viruses. In the present study, we analyzed multi-year surveillance data from Yunnan to examine the evolutionary characteristics, antigenic drift, and antiviral susceptibility of the currently circulating influenza strains. Additionally, we assessed the efficacy of the A/H3N2 component vaccine recommended by the WHO for the Northern Hemisphere against the strains circulating in Yunnan.

## 2. Materials and Methods

### 2.1. Specimen Collection and Isolation/Identification of Viruses

Patients exhibiting influenza-like illness (ILI) who were admitted to sentinel hospitals in Yunnan Province, China between 1 January 2017 and 31 December 2022 were monitored. The ILI was characterized by a sudden onset of high fever (≥38 °C) accompanied by respiratory symptoms (e.g., cough and sore throat). Samples were collected with informed consent from the patients or their guardians. Throat swabs, preserved in viral transport medium, were sent to Yunnan influenza surveillance network laboratories within 24 h for further influenza virus detection through RNA extraction using an automatic preparation system (Tianlong NP968, Xian, China) and real-time reverse transcription polymerase chain reaction (RT-PCR) using FluA-Forward: 5′-GACCRATCC TGTCACCTCTGAC-3′, FluA-Reverse: 5′-GGGCATTYTGGACAAAKCGTCTACG-3′, FluA-Probe: 5′-TGCAGTCCTCGCTCACTGGGCACG-3′, H3-Forward: 5′-ACCCTCAGTGTGATGGCTTTCAAA-3′, H3-Reverse: 5′-TAAGGGAGGCATAACCCGGCACAT-3′, and H3-Probe: 5′-AMGAAGCARAGCCTACAGCAGCTGT-3′. Next, 1–2 mL positive throat swab samples were inoculated with Madin-Darby canine kidney (MDCK) cells obtained from the Chinese National Influenza Center in cell culture bottle T25 (Corning) and incubated at 35 °C and 5% CO_2_ for 1-2 h. After incubation, the cells were washed with Phosphate Buffer Saline (PBS, Gibco, 20012050), and then 5 mL of Dulbecco’s Modified Eagle’s Medium (Gibco, 11965118) containing 100 U/mL of penicillin, 100 μg/mL of streptomycin, and 2 μg/mL of TPCK-trypsin were added to cell culture bottle T25 and cultured in 35 °C and 5% CO_2_. The cytopathic changes were observed for 4–7 days to collect the viruses, which were subsequently confirmed through hemagglutination assay. The test virus was twofold serially diluted with PBS in a volume of 50 μL/well in duplicate. Then, 50 microliters of 1% guinea pig erythrocyte suspended in PBS was added to the test well and incubated at room temperature for 30–45 min to determine the hemagglutination results. The analysis of the influenza subtypes was carried out using a hemagglutination inhibition assay with post-infection ferret antisera raised against annual recommended Northern Hemisphere vaccine strains (2017–2022) from the Chinese National Influenza Center (Beijing, China). And, specific experimental procedures refer to the Technical Guidelines for National Influenza Surveillance (2017 Edition) (https://ivdc.chinacdc.cn/cnic/fascc/201802/t20180202_158592.htm, accessed on 30 September 2017). The identification results were reviewed by the Yunnan Center for Disease Control and Prevention before being forwarded to the Chinese National Influenza Center for final confirmation.

### 2.2. Whole-Genome Sequencing of Influenza A/H3N2 Viruses

Nucleic acids from the isolated strains were extracted using an automatic nucleic acid extractor (BioPerfectus SAW-96, Jiangsu, China) and utilized for cDNA synthesis and PCR amplification employing the SuperScript^TM^ III One-Step RT-PCR System with Platinum^TM^ *Taq* High Fidelity (Invitrogen, Carlsbad, CA, USA, 12574035). The amplification primers included Uni-12/Inf1 (primer A): 5′-GGGGGGAGCAAAAGCAGG-3′, Uni-12/Inf3 (primer B): 5′-GGGGGGAGCGAAAGCAGG-3′ and Uni-13/Inf1 (primer C): 5′-CGGGTTATTAGTAGAAACAAGG-3′. The preparation of the reaction system and conditions is detailed in the Technical Guidelines for National Influenza Surveillance (2017 Edition). The resulting amplification products were purified using magnetic beads (Beckman, A63881), quantified using a Qubit^TM^ dsDNA HS Assay Kit (Invitrogen, Q32854), and diluted to 0.2 ng/μL. Sequencing libraries were then prepared using a Nextera XT DNA Library Prep kit (Illumina, FC-131-1096) according to the provided instructions. Finally, the libraries were pooled, denatured, diluted to 13pM, and loaded onto the reagent cartridge (MiSeq v2 Reagent Tray 300 cycles-PE) in the designated reservoir and sequenced on the Illumina MiSeq Sequencing Platform per the Illumina MiSeq system guide. Raw paired-end reads were imported into CLC Genomics Workbench 22 (Qiagen, Aarhus, Denmark) and further trimmed based on quality scores ≥ 0.05 and ambiguous nucleotides ≤ 2, with the 5′ and 3′ sequencing adaptors trimmed by removing 5 and 18 terminal nucleotides, respectively. Reference-based data analyses were performed in this study. High-quality sequencing reads were then aligned to the vaccine strains of the corresponding year using the parameters: match score = 1; mismatch cost = 2 (linear gap cost); length fraction = 0.5; similarity fraction = 0.8; auto-detect paired distance; non-specific match handling: map randomly. Consensus sequences with a sequencing depth ≥ 3300X were subsequently generated for downstream analyses. In this study, all 253 strain names and their corresponding accession numbers are listed in the attachment material (Table S1).

### 2.3. Phylogenetic Analyses of Influenza A/H3N2 Viruses

Twenty-two global reference strains, including six Northern Hemisphere vaccine strains (2017–2022) recommended by WHO, were retrieved from the Global Initiative on Sharing All Influenza Data (GISAID) and NCBI databases (Table S1). Multiple sequence alignment was performed using MAFFT (v7) (https://mafft.cbrc.jp/alignment/server/index.html, accessed on 6 September 2017), with default parameters. RAxML (v7) [18] was then applied to construct the evolutionary phylogeny using the GTRGAMMA model with a bootstrap value of 1000. The best phylogenetic trees were visualized using FigTree (v1.4.3) (http://tree.bio.ed.ac.uk/software/figtree/, accessed on 4 October 2016).

### 2.4. Sequence Homologies and Mutation Analyses of Influenza A/H3N2 Viruses

The divergence of nucleotide sequences in influenza A/H3N2 viruses was determined using pair-wise nucleotide sequence identities of annually circulating strains with the Basic Local Alignment Search Tool algorithm [19]. This analysis focused specifically on the HA and NA gene segments of the influenza A/H3N2 viruses in Yunnan. Mutation analyses at the amino acid level were performed on all eight gene segments using MEGA X [20] and compared to the reference strain A/Texas/50/2012.2.5. 

### 2.5. Selective Constraint Analyses of Influenza A/H3N2 Viruses

The non-synonymous/synonymous substitution ratio (d_N_/d_S_) was considered when evaluating codons under selective pressure. Analyses of the d_N_/d_S_ ratios across all eight gene segments were conducted using single likelihood ancestor counting (SLAC), mixed effects model of evolution (MEME), and fixed effects likelihood (FEL) methods. All algorithms were implemented in HYPHY, accessible on the Datamonkey webserver (https://www.datamonkey.org/, accessed on 2 January 2018). Positively selected sites were identified based on statistical significance (*p*-value < 0.1).

### 2.6. Vaccine Effectiveness Evaluation

Estimated vaccine effectiveness (VE) against the influenza A/H3N2 viruses was determined using the P_epitope_ model, which evaluates the antigenic distance between the vaccine and circulating strains [17] based on the proportion of substituted amino acid residues in the dominant HA epitope. Notably, P_epitope_ is calculated by dividing the number of amino acid substitutions on the HA1 epitope by the total number of amino acids on the epitope. The amino acid residues on the HA epitope of A/H3N2 viruses were pre-defined in the P_epitope_ calculator with 19, 21, 27, 41, and 22 amino acids. The relationship between VE and P_epitope_ is described by the formula VE = −2.47 × P_epitope_ + 0.47, where VE is 47% when P_epitope_ = 0 [21].

The whole genomes of the virus strains circulating each year were compared to the Northern Hemisphere vaccine strains of the corresponding years, and mutation analyses were conducted using MEGA X. The strains collected from 2017 to 2022 were compared to A/Hong Kong/4801/2014, A/Singapore/INFIMH-16-0019/2016, A/Kansas/14/2017, A/Hong Kong/45/2019, A/Cambodia/e0826360/2020, and A/Darwin/6/2021, separately.

## 3. Results

### 3.1. Isolation Rate and Demographic Characteristics of Influenza

In this study, 136,996 samples from patients exhibiting influenza-like illness were collected through sentinel hospital surveillance in Yunnan from 1 January 2017 to 31 December 2022. Among these samples, 5091 strains were isolated, with influenza A and B strains accounting for 3279 (2.39%) and 1812 (1.32%), respectively. Specifically, 1372 (41.84%) were classified as A/H3N2 and 1907 (58.16%) were classified as A/H1N1 (Table S2). Of the 5091 cases infected with influenza, a majority were males (55.86%), and infections primarily occurred in the 0 to 14 years age group, with infants and adolescents being most affected (Table S3), consistent with a previous study [22].

**Figure 1:**
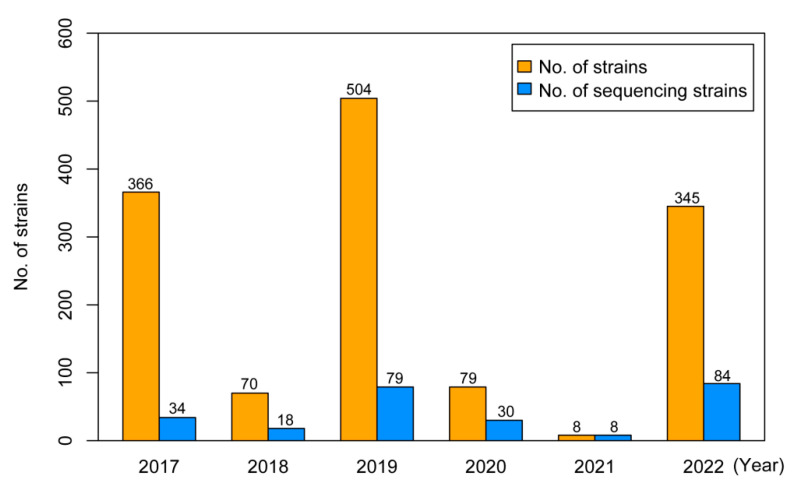
Sample distribution of total and sequenced strains of influenza A/H3N2 viruses in Yunnan from 2017 to 2022. The number each year is also marked.

In total, 253 strains were randomly selected from 1372 isolated A/H3N2 strains in Yunnan to perform whole-genome sequencing analyses. The sampling distribution of selected strains is plotted in Figure 1. On average, the sequencing strains represented approximately 35.63% of the total A/H3N2 strains isolated each year.

### 3.2. Phylogenetic Patterns Highlight High Sequence Diversity of Influenza A/H3N2 Viruses in 2018

**Figure 2:**
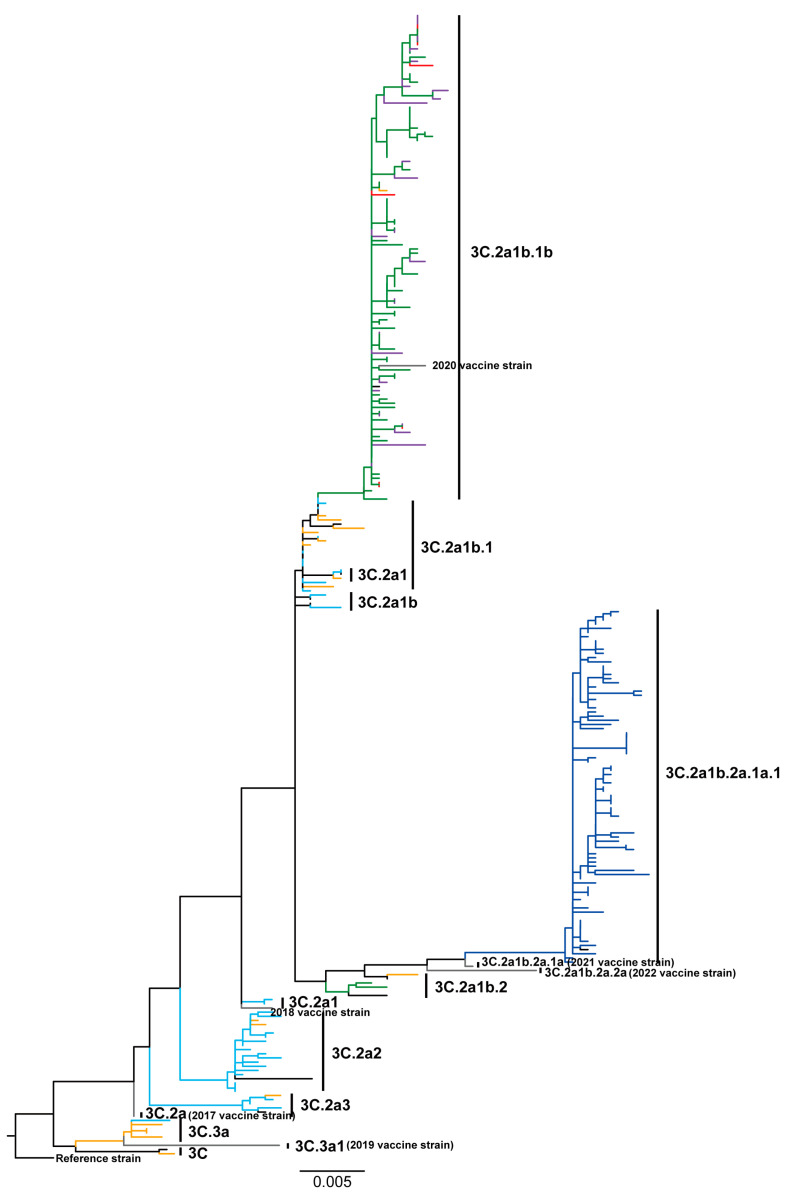
Phylogenetic analyses of HA segments of influenza A/H3N2 viruses in Yunnan Province, China, from 2017 to 2022. Ruler value (0.005) represents genetic distance. The 2017 Yunnan strains: DeepSkyBlue (#00BFFF); the 2018 Yunnan strains: Orange (#FFA500); the 2019 Yunnan strains: ForestGreen (#228B22); the 2020 Yunnan strains: Purple (#A020F0); the 2021 Yunnan strains: Red (#FF0000); the 2022 Yunnan strains: Blue (#0000FF); vaccine strains: Grey; the reference strain: Black.

Phylogenetic analysis of HA showed that most (16/34) of the 2017 Yunnan sequences were clustered in clade 3C.2a2. The evolutionary clades of the remaining 2017 Yunnan sequences were as follows: 3C.2a1b.1 (9/34), 3C.2a1 (3/34), 3C.2a3 (3/34), 3C.2a1b (2/34), and 3C.3a (1/34) (Figure 2). None of the 2017 Yunnan sequences clustered with the 2017 vaccine strain A/Hong Kong/4801/2014 (3C.2a). The clades of 2018 Yunnan sequences were as follows: 3C.2a1b.1 (7/18), 3C.3a (4/18), 3C.2a2 (2/18), 3C (1/18), 3C.2a1 (1/18), 3C.2a3 (1/18), 3C.2a1b.1b (1/18), and 3C.2a1b.2 (1/18). Hence, the 2018 Yunnan circulating strains exhibited a high evolutionary distance to the 2018 vaccine strain A/Singapore/INFIMH-16-0019/2016 (3C.2a1). Almost all (76/79) of the 2019 Yunnan sequences were clustered in clade 3C.2a1b.1b, distant from the 2019 vaccine strain A/Kansas/14/2017 (3C.3a1). All 2020 Yunnan sequences were clustered in clade 3C.2a1b.1b, including the 2020 vaccine strain A/Hong Kong/45/2019. All 2021 Yunnan sequences were also clustered in clade 3C.2a1b.1b, detached from the 2021 vaccine strain A/Cambodia/e0826360/2020 located in clade 3C.2a1b.2a.1a. All 2022 Yunnan sequences were clustered in clade 3C.2a1b.2a.1a.1, with substantial evolutionary divergence from the 2022 vaccine strain A/Darwin/6/2021 (3C.2a1b.2a.2a).

**Figure 3:**
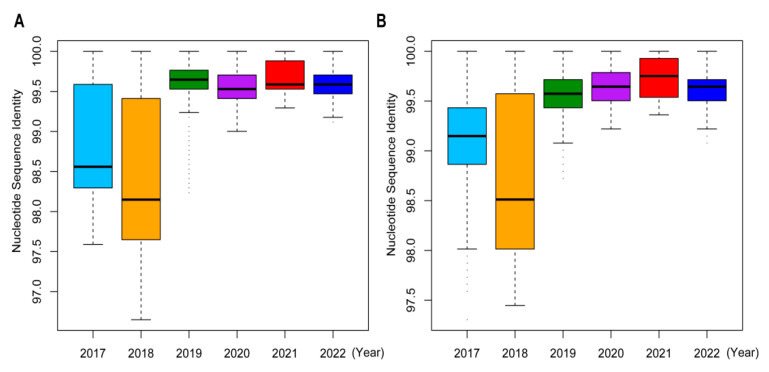
Sequence homology analyses of HA and NA segments of influenza A/H3N2 viruses in Yunnan Province, China, from 2017 to 2022. (**A**) Sequence identity comparisons of HA segments across 2017–2022. (**B**) Sequence identity comparisons of NA segments across 2017–2022.

Analysis of HA phylogeny revealed higher branch heterogeneity for Yunnan strains from 2017 to 2018 than those from 2019 to 2022. The HA sequence homologies for the Yunnan strains were lowest in 2018 and 2017 (Figure 3A). Moreover, the 2017–2018 strains harbored significantly higher diversity compared to the 2019–2022 strains, as evidenced by their lower nucleotide sequence identities of NA segments (Figure 3B). These findings underscored the diversity and complexity of influenza A/H3N2 viruses, especially in 2017 and 2018. Our analyses also revealed a consistent mismatch between the vaccine and circulating strains almost every year. Notably, the evolutionary patterns for the seven other gene segments, including NA, M, NP, NS, PA, PB1, and PB2, were consistent with those of HA (Figures S1–S7).

### 3.3. Amino Acid Mutation Analyses of Influenza A/H3N2 Viruses

**Figure 4:**
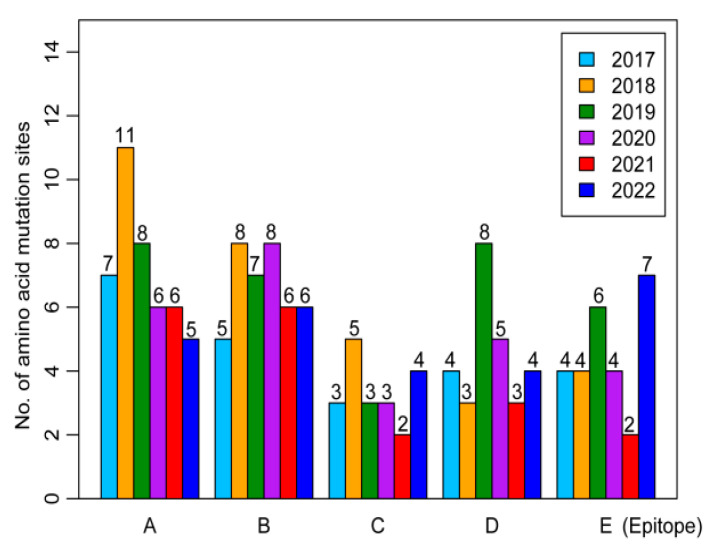
Evolution of HA antigenic epitopes for influenza A/H3N2 viruses in Yunnan Province, China, from 2017 to 2022. Detailed analysis of amino acid mutation sites within epitopes A, B, C, D, and E was conducted.

Evolutionary heterogeneity was observed in the HA antigenic epitopes of influenza A/H3N2 viruses from 2017 to 2022 relative to the reference strain A/Texas/50/2012. In the Yunnan 2017–2018 strains, antigenic epitope A harbored the most mutation sites compared to the B, C, D, and E epitopes. For the 2019 strains, epitopes A and D exhibited the most mutation sites, while B epitope contained the most mutation sites in the 2020 strains, epitopes A and B possessed the largest mutation sites in the 2021 strains, and epitope E incorporated the majority of mutation sites in the 2022 strains (Figure 4 and Table S4).

Compared to the reference strain A/Texas/50/2012, a total of 17 amino acid substitutions (L3I, E62G, K92R, N121K, N128T/A, R142G, N144S, N145S, F159Y, K160T, V186G/S, F193S, F219S, N225D, I406V, G484E, and D489N) were detected in the HA proteins of most influenza A/H3N2 strains (2017–2022) (Table S4). Among these mutations, five (L3I, N144S, N145S, F159Y, and K160T) were previously reported in Myanmar from 2015 to 2019 [16]. Additionally, five new mutations (I48T, K83E, Y94N, Y195F, and I522M) were dominant in the Yunnan 2022 strains. In total, 12 mutations were located in the antigenic epitopes, including E62G and K92R in epitope E; N121K and F219S in epitope D; N128T/A, F159Y, K160T, V186G/S, and F193S in epitope B; and R142G, N144S, and N145S in epitope A. Notably, the F159Y and K160T substitutions, along with an existing N at site 158, a potential glycosylation site, may hinder viral epitopes and antibody access to the antigenic sites [23].

The NA protein promotes the release of viral particles from host cells via enzymatic activity [8]. Certain mutations in the NA protein (E41G, E119V/I/D, Q136K, T148I, D151A/E/G, I222L, R224K, N245Y, deletion 245–248, deletion 247–250, K249E, E276D, R292K, N294S, N329K, S331R, R371K, and Q391K) can lead to resistance to neuraminidase inhibitors, such as oseltamivir, zanamivir, laninamivir, and peramivir [24,25]. Here, we analyzed all 253 Yunnan strains to detect the frequency of drug-resistant amino acid mutations of the NA protein. Only a few strains from 2017, 2019, and 2022 exhibited amino acid substitutions, potentially associated with resistance characteristics (Table S5). For example, one 2017 strain carried the amino acid mutation K249E, which reduces susceptibility to oseltamivir [26]. Another 2017 strain contained the amino acid substitution N329K, which confers resistance to oseltamivir and zanamivir [27]. A 2019 strain covered the amino acid substitution E41G, which could reduce the susceptibility to oseltamivir [28]. The amino acid substitution T148I, related to a decrease in susceptibility to zanamivir, was observed in one 2022 strain [29].

All 253 Yunnan strains potentially exhibited resistance to amantadine, as proven by the S31N amino acid substitution in the M2 protein (Table S5). They also shared a common substitution R384G in the NP protein (Table S5), and this mutation may exert an influence on viral fitness [30]. The substitution K196E in NS1 protein, associated with heightened virulence and increased disease severity [31], was found in one 2019 strain (Table S5). The V668I mutation in the PA protein, which may attenuate viral temperature sensitivity and enhance viral replication [32], was present in the majority of Yunnan strains (90.12%) during 2017–2022 (Table S5). The PB1-F2 protein, known for its pro-apoptotic role localized in mitochondria, is involved in promoting inflammation and regulating viral polymerase activity [33,34,35]. Analysis of the PB1-F2 open reading frames (ORFs) indicated that the majority (23/34) of the 2017 sequences possessed the full-length PB1-F2 of 90 aa, while a smaller portion (9/34) had a variant with 87 aa (Table S5). The remaining two 2017 sequences had a truncated PB1-F2 protein with 57 aa. In 2018, an 87 aa truncation of PB1-F2 was predominant, accounting for 55.56% of all sequences, which increased to 99% from 2019 to 2022. A single 2020 and a single 2022 sequence produced PB1-F2 truncations of 57 and 24 aa, respectively. Pimodivir impacts early replication of influenza A virus by inhibiting PB2 cap-binding, and the PB2 mutations S324C/R or N510K could reduce its efficacy [36]. However, these specific PB2 substitutions were not detected in this study.

### 3.4. Selection Pressure Analyses of Influenza A/H3N2 Viruses

To assess the selective constraint on protein-coding sequences, the overall d_N_/d_S_ ratio was calculated using SLAC [37]. The results showed that M2 and NS1 experienced rapid evolution, with the highest overall d_N_/d_S_ values of 0.514 and 0.455, respectively (Table 1). The HA, NA, and NS2 proteins showed moderate overall d_N_/d_S_ values of 0.258, 0.268, and 0.260, separately. The overall d_N_/d_S_ values for the M1, PA, PB1, and PB2 proteins ranged from 0.022 to 0.088, suggesting strong purifying selection.

**Figure 5:**
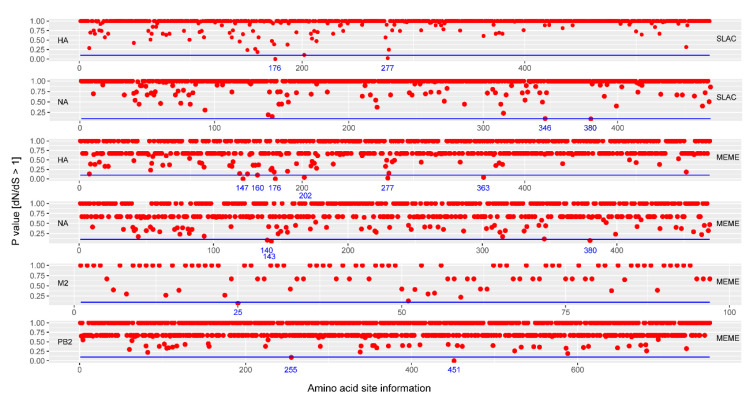
Screening information of potential positively selected sites of influenza A/H3N2 viruses using SLAC and MEME algorithms. Here, the specific screening information from the FEL algorithm was excluded in this figure due to limited outputting information according this method. The blue line represents the cutoff of 0.1 (*p*-value).

During infection, amino acids on coding sequences often experience changes due to host immune pressure on the virus [38]. Therefore, we used three reliable algorithms (SLAC, MEME, and FEL) to further evaluate potential positively selected sites [37,39] (Figure 5). SLAC identified strong positive selection in HA (176 and 277) and NA (346 and 380) (*p* < 0.02, Table 1). Moreover, MEME revealed positively selected sites in HA (147, 160, 176, 202, 277, and 363), NA (140, 143, and 380), M2 (25), and PB2 (255 and 451), respectively. Meanwhile, FEL indicated three positively selected sites in HA (176, 202, and 277), four positively selected sites in NA (140, 143, 346, and 380), one positively selected site in M2 (25), and one positively selected site in PB2 (255).

Integrally, our analyses disclosed key positively selected sites in HA (176, 202, and 277), NA (140, 143, 346, and 380), M2 (25), and PB2 (255). These sites likely contribute to the adaptive evolution of influenza A/H3N2 strains in Yunnan, helping them respond to host immune defenses.

### 3.5. Estimation of Vaccine Efficacy for Influenza A/H3N2 Viruses

Amino acid residues in five epitopes (A–E), which possess 19, 21, 27, 41, and 22 amino acids, respectively, were defined according to prior research [17,21]. The predicted vaccine efficacies of various vaccine strains against Yunnan 2017–2022 strains were also evaluated in this study (Table 2 and Table S6).

For the Yunnan strains in 2017 and 2018, the P_epitope_ against vaccine strains A/Hong Kong/4801/2014 and A/Singapore/INFIMH-16-0019/2016 was 0.1053 (dominant epitope A), suggesting a predicated vaccine efficacy of 21%. For 2019, antigenic drift mainly in epitope E of the HA sequences led to a P_epitope_ of 0.1364, indicating a decreased vaccine efficacy of 13.32% for A/Kansas/14/2017. For 2020, the amino acid substitutions dominated on epitope C, yielding a vaccine efficacy of 37.85% for A/Hong Kong/45/2019. Compared with the vaccine strain A/Cambodia/e0826360/2020, the predicated vaccine efficacy against Yunnan 2021 strains exhibited −5.00%, suggesting a visibly descent. For 2022 strains, a P_epitope_ of 0.2381 (dominant epitope B) supported a further decline in vaccine efficacy (−11.81%) in contrast with the vaccine strain A/Darwin/6/2021.

## 4. Discussion

This study aimed to better understand evolutionary characteristics of circulating influenza A/H3N2 strains in Yunnan Province, China from 2017 to 2022.

We observed that the influenza A/H3N2 strains isolated from 2017 to 2018 in Yunnan exhibited higher genetic diversity, reflected in a broader distribution across evolutionary clades, compared to the 2019–2022 strains. This trend aligns with previous influenza surveillance studies [40,41,42]. However, from 2019 to 2022, the evolution of the Yunnan strains showed relatively stability with a high nucleotide sequence homology, predominantly clustering in the 3C.2a1b.1b clade for 2019–2021 and the 3C.2a1b.2a.1a.1 clade for 2022. This pattern diverges markedly from Myanmar circulating strains in 2019 (3C.2a1b) and 2021 (3C.2a1b.2a.2a.3) and Italy/Australia circulating strains in 2021 and 2022 (3C.2a1b.2a.2) [16,43,44,45].

The HA protein is crucial in influenza virus virulence and antigenic variations [9]. Intriguingly, our results demonstrated greater heterogeneity in the evolution of antigenic epitopes of influenza A/H3N2 strains from 2017 to 2022 compared with the A/Texas/50/2012 reference strain, highlighting a refined evolutionary strategy of these local influenza A/H3N2 strains in Yunnan. Previous studies suggest that four or more simultaneous amino acid mutations in two or more antigenic epitopes could predict an epidemiological antigenic drift [46,47]. In most of the Yunnan 2017–2022 strains, 12 mutations were identified in four epitope regions (A, B, D, and E) of the HA protein, potentially altering the antigenicity of influenza A/H3N2 strains circulating in Yunnan. Therefore, continuous monitoring of amino acid substitutions accumulating in the antigenic sites of the HA proteins of influenza A/H3N2 viruses is essential. In addition, the majority of the Yunnan 2022 strains originated five new mutations (I48T, K83E, Y94N, Y195F, and I522M) in the HA protein, emphasizing the need for future functional studies on these variations.

Most Yunnan 2017–2022 strains were sensitive to neuraminidase inhibitors, although several mutations associated with drug resistance on the NA protein of a few strains merit further monitoring and attention. However, the Yunnan 2017–2022 strains were resistant to amantadine, as reported in previous studies [23,48,49]. Almost all Yunnan strains harbored a full-length PB1-F2 protein (87 or 90aa), consistent with other research [50], although four strains contained the truncated PB1-F2 proteins (57 or 24aa). Nevertheless, at present, the impact of these truncations on the infectivity and transmissibility of influenza A/H3N2 viruses remains unclear and needs to be functionally validated in future research.

The process of host–pathogen interactions drives ongoing selection pressure for viral adaptation [51]. Here, the accelerated genomic evolution of Yunnan strains from 2017 to 2022 was primarily reflected in the M2, NS1, NA, NS2, and HA segments, showing higher overall d_N_/d_S_ values, in basic alignment with previous studies [52,53]. By utilizing diverse algorithms, we found that two amino acid sites (176 and 277) in HA experienced strong positive selection. Importantly, the residue 176 was located in antigenic epitope D, which plays a pivotal role in antigenic determinants and the emergence of novel variant strains [10].

We further predicted the vaccine efficacy of the Yunnan 2017–2022 strains. The results showed a slight decline in predicted vaccine efficacy in 2017 and 2018, as reported in earlier research [41,42]. Conversely, when the vaccine strain was switched to the A/Hong Kong/45/2019 in 2020, the vaccine efficacy displayed a significant improvement. Meanwhile, the modifications made to the vaccine components targeting A/H3N2 viruses for 2021–2022 failed to provide even marginal protection against the prevailing A/H3N2 strains in Yunnan, as evidenced by a decline in vaccine efficacy to negative values. Given that the recommended vaccine strain for the Northern Hemisphere in 2022–2023 remains A/Darwin/6/2021 (belonging to the 3C.2a1b.2a.2 clade), should the circulating strains in Yunnan continue to evolve within the 3C.2a1b.2a.1a.1 clade, it is highly probable that this vaccine component will prove inadequate in conferring improved vaccine protection against the Yunnan circulating strains in 2023. Thus, our study underscores the importance of ongoing surveillance of complete influenza virus genomes, thereby facilitating the identification of potential candidate vaccine strain lineages and enhancing our capacity to anticipate the strains that may persist in subsequent years.

In conclusion, our study uncovers the evolutionary features of influenza A/H3N2 viruses in Yunnan from 2017 to 2022 and further highlights the decline of vaccine efficacy against the circulating A/H3N2 strains in Yunnan, especially during the 2021–2022 period. Our findings also underscore the importance of ongoing surveillance and molecular profiling of influenza viruses.

## Figures and Tables

**Table 1:** Selection pressures and positively selected sites of coding sequences of influenza A/H3N2 viruses.

Coding Region	SLAC (Single-Likelihood Ancestor Counting)			MEME (Mixed Effects Model of Evolution)			FEL (Fixed Effects Likelihood)		
	d_N_*/*d_S_	Position	*p*-Value	d_N_*/*d_S_	Position	*p*-Value	d_N_*/*d_S_	Position	*p*-Value
PB2	0.079	N/D	N/D	0.075	255	0.090	0.079	255	0.065
					451	0.000			
PB1	0.084	N/D	N/D	0.078	N/D	N/D	0.084	N/D	N/D
PA	0.088	N/D	N/D	0.081	N/D	N/D	0.088	N/D	N/D
HA	0.258	176	0.000	0.228	147	0.000	0.258	176	0.000
		277	0.023		160	0.100		202	0.075
					176	0.000		277	0.011
					202	0.040			
					277	0.020			
					363	0.040			
NP	0.068	N/D	N/D	0.063	N/D	N/D	0.063	N/D	N/D
NA	0.268	346	0.099	0.235	140	0.080	0.268	140	0.060
		380	0.091		143	0.050		143	0.036
					380	0.070		346	0.087
								380	0.053
M1	0.022	N/D	N/D	0.020	N/D	N/D	0.020	N/D	N/D
M2	0.514	N/D	N/D	0.483	25	0.070	0.483	25	0.053
NS1	0.455	N/D	N/D	0.445	N/D	N/D	0.445	N/D	N/D
NS2	0.260	N/D	N/D	0.243	N/D	N/D	0.243	N/D	N/D

Note: d_N_/d_S_, non-synonymous/synonymous substitution ratio; *p*-value of the SLAC, MEME, and FEL results showing positive selection level; N/D, not detected.

**Table 2:** Efficacy estimation and dominant epitope mutation analysis of influenza A/H3N2 strains in Yunnan (2017–2022).

Year (N)	Vaccine Strain	No. of Strains	Dominant Epitope	No. of Mutations	Residue Differences	P_epitope_	Vaccine Efficacy (%)
2017 (N = 34)	A/Hong Kong/4801/2014	16	A	2	T131K, R142K	0.1053	21.00
2018 (N = 18)	A/Singapore/INFIMH-16-0019/2016	10	A	1	T135K/N,	0.0526	34.00
		1	A	2	S144K, R150K	0.1053	21.00
2019 (N = 79)	A/Kansas/14/2017	79	E	3	E62G, N91S, K92R	0.1364	13.32
2020 (N = 30)	A/Hong Kong/45/2019	30	C	1	N312S	0.0370	37.85
2021 (N = 8)	A/Cambodia/e0826360/2020	8	A	4	K131T, T135K, S137F, A138S	0.2105	−5.00
2022 (N = 84)	A/Darwin/6/2021	84	B	5	S156H, N159Y, D186S, N190D, S198P	0.2381	−11.81

## Data Availability

Data are contained within the article and supplementary materials.

## Supplementary Materials

The following supporting information can be downloaded at: https://www.mdpi.com/article/10.3390/v16010138/s1, Figures S1–S7: Phylogenetic analyses of NA (Figure S1), M (Figure S2), NP (Figure S3), NS (Figure S4), PA (Figure S5), PB1 (Figure S6), and PB2 (Figure S7) segments of influenza A/H3N2 viruses in Yunnan Province, China, from 2017 to 2022. Ruler value (0.003, 0.002, 0.003, 0.003, 0.003, 0.003 and 0.003) respectively represents genetic distance for each segment. The 2017 Yunnan strains: DeepSkyBlue (#00BFFF); the 2018 Yunnan strains: Orange (#FFA500); the 2019 Yunnan strains: ForestGreen (#228B22); the 2020 Yunnan strains: Purple (#A020F0); the 2021 Yunnan strains: Red (#FF0000); the 2022 Yunnan strains: Blue (#0000FF); vaccine strains: Grey; the reference strain: Black; Table S1: Strain names and accession numbers of 8 segments of influenza A/H3N2 viruses isolated in Yunnan Province, China, from 2017 to 2022, and the 22 global reference strains; Table S2: The number (%) of influenza strains isolated from samples of influenza-like cases in Yunnan Province, China, from 2017 to 2022; Table S3: Demographic characteristics of the cases infected with influenza viruses in Yunnan Province, China, from 2017 to 2022; Table S4: Amino acid mutation analyses of HA proteins for influenza A/H3N2 strains in Yunnan Province, China, from 2017 to 2022; Table S5: Key mutations of other protein sequences and protein length of PB1-F2 for influenza A/H3N2 strains in Yunnan Province, China, from 2017 to 2022; Table S6: Efficacy estimation among influenza A/H3N2 vaccine strains and mutations found on the dominant epitopes of influenza A/H3N2 strains in Yunnan Province, China, from 2017 to 2022.
