# Supplementary material for: Genomic Analyses Uncover Evolutionary Features of Influenza A/H3N2 Viruses in Yunnan Province, China, from 2017 to 2022
Source: Viruses. 2024 Jan 18;16(1):138. doi: 10.3390/v16010138 (PMC10820241; doi:10.3390/v16010138)
Supplement: Supplementary file 1 [file viruses-16-00138-s001.zip › Table S1.pdf]

Table S1. Strain names and accession numbers of eight segments of influenza A/H3N2 viruses isolated in Yunnan Province, China, from 2017 to 2022, and the 22 global reference strains.

Strain name	ID	PB2	PB1	PA	HA	NP	NA	M	NS
A/Yunnan-Guandu/1669/2017	EPI_ISL_18681104	EPI2864047	EPI2864048	EPI2864046	EPI2864050	EPI2864043	EPI2864049	EPI2864045	EPI2864044
A/Yunnan-Dali/1392/2017	EPI_ISL_18681105	EPI2864055	EPI2864056	EPI2864054	EPI2864058	EPI2864051	EPI2864057	EPI2864053	EPI2864052
A/Yunnan-Dali/1395/2017	EPI_ISL_18681106	EPI2864063	EPI2864064	EPI2864062	EPI2864066	EPI2864059	EPI2864065	EPI2864061	EPI2864060
A/Yunnan-Lushui/1893/2017	EPI_ISL_18681107	EPI2864071	EPI2864072	EPI2864070	EPI2864074	EPI2864067	EPI2864073	EPI2864069	EPI2864068
A/Yunnan-Chuxiong/1363/2017	EPI_ISL_18681108	EPI2864079	EPI2864080	EPI2864078	EPI2864082	EPI2864075	EPI2864081	EPI2864077	EPI2864076
A/Yunnan-Xianggelila/1381/2017	EPI_ISL_18681109	EPI2864087	EPI2864088	EPI2864086	EPI2864090	EPI2864083	EPI2864089	EPI2864085	EPI2864084
A/Yunnan-Xishan/1748/2017	EPI_ISL_18681110	EPI2864095	EPI2864096	EPI2864094	EPI2864098	EPI2864091	EPI2864097	EPI2864093	EPI2864092
A/Yunnan-Wenshan/1727/2017	EPI_ISL_18681111	EPI2864103	EPI2864104	EPI2864102	EPI2864106	EPI2864099	EPI2864105	EPI2864101	EPI2864100
A/Yunnan-Wenshan/1863/2017	EPI_ISL_18681112	EPI2864111	EPI2864112	EPI2864110	EPI2864114	EPI2864107	EPI2864113	EPI2864109	EPI2864108
A/Yunnan-Hongta/11239/2017	EPI_ISL_18681113	EPI2864119	EPI2864120	EPI2864118	EPI2864122	EPI2864115	EPI2864121	EPI2864117	EPI2864116
A/Yunnan-Mengzi/1824/2017	EPI_ISL_18681114	EPI2864127	EPI2864128	EPI2864126	EPI2864130	EPI2864123	EPI2864129	EPI2864125	EPI2864124
A/Yunnan-Linxiang/11042/2017	EPI_ISL_18681115	EPI2864135	EPI2864136	EPI2864134	EPI2864138	EPI2864131	EPI2864137	EPI2864133	EPI2864132
A/Yunnan-Xianggelila/1728/2017	EPI_ISL_18681116	EPI2864143	EPI2864144	EPI2864142	EPI2864146	EPI2864139	EPI2864145	EPI2864141	EPI2864140
A/Yunnan-Simao/1948/2017	EPI_ISL_18681117	EPI2864151	EPI2864152	EPI2864150	EPI2864154	EPI2864147	EPI2864153	EPI2864149	EPI2864148
A/Yunnan-Hongta/1879/2017	EPI_ISL_18681118	EPI2864159	EPI2864160	EPI2864158	EPI2864162	EPI2864155	EPI2864161	EPI2864157	EPI2864156
A/Yunnan-Mengzi/1732/2017	EPI_ISL_18681119	EPI2864167	EPI2864168	EPI2864166	EPI2864170	EPI2864163	EPI2864169	EPI2864165	EPI2864164
A/Yunnan-Linxiang/11037/2017	EPI_ISL_18681120	EPI2864175	EPI2864176	EPI2864174	EPI2864178	EPI2864171	EPI2864177	EPI2864173	EPI2864172
A/Yunnan-Dali/1386/2017	EPI_ISL_18681121	EPI2864183	EPI2864184	EPI2864182	EPI2864186	EPI2864179	EPI2864185	EPI2864181	EPI2864180
A/Yunnan-Mengzi/11020/2017	EPI_ISL_18681122	EPI2864191	EPI2864192	EPI2864190	EPI2864194	EPI2864187	EPI2864193	EPI2864189	EPI2864188
A/Yunnan-Wenshan/1620/2017	EPI_ISL_18681123	EPI2864199	EPI2864200	EPI2864198	EPI2864202	EPI2864195	EPI2864201	EPI2864197	EPI2864196
A/Yunnan-Hongta/11430/2017	EPI_ISL_18681124	EPI2864207	EPI2864208	EPI2864206	EPI2864210	EPI2864203	EPI2864209	EPI2864205	EPI2864204
A/Yunnan-Hongta/1739/2017	EPI_ISL_18681125	EPI2864215	EPI2864216	EPI2864214	EPI2864218	EPI2864211	EPI2864217	EPI2864213	EPI2864212

A/Yunnan-Mengzi/1699/2017	EPI_ISL_18681126	EPI2864223	EPI2864224	EPI2864222	EPI2864226	EPI2864219	EPI2864225	EPI2864221	EPI2864220
A/Yunnan-Anning/1595/2017	EPI_ISL_18681127	EPI2864231	EPI2864232	EPI2864230	EPI2864234	EPI2864227	EPI2864233	EPI2864229	EPI2864228
A/Yunnan-Hongta/11304/2017	EPI_ISL_18681128	EPI2864239	EPI2864240	EPI2864238	EPI2864242	EPI2864235	EPI2864241	EPI2864237	EPI2864236
A/Yunnan-Dali/1380/2017	EPI_ISL_18681129	EPI2864247	EPI2864248	EPI2864246	EPI2864250	EPI2864243	EPI2864249	EPI2864245	EPI2864244
A/Yunnan-Hongta/11114/2017	EPI_ISL_18681130	EPI2864255	EPI2864256	EPI2864254	EPI2864258	EPI2864251	EPI2864257	EPI2864253	EPI2864252
A/Yunnan-Linxiang/11041/2017	EPI_ISL_18681131	EPI2864263	EPI2864264	EPI2864262	EPI2864266	EPI2864259	EPI2864265	EPI2864261	EPI2864260
A/Yunnan-Linxiang/1611/2017	EPI_ISL_18681132	EPI2864271	EPI2864272	EPI2864270	EPI2864274	EPI2864267	EPI2864273	EPI2864269	EPI2864268
A/Yunnan-Linxiang/11036/2017	EPI_ISL_18681133	EPI2864279	EPI2864280	EPI2864278	EPI2864282	EPI2864275	EPI2864281	EPI2864277	EPI2864276
A/Yunnan-Linxiang/1507/2017	EPI_ISL_18681134	EPI2864287	EPI2864288	EPI2864286	EPI2864290	EPI2864283	EPI2864289	EPI2864285	EPI2864284
A/Yunnan-Linxiang/1616/2017	EPI_ISL_18681135	EPI2864295	EPI2864296	EPI2864294	EPI2864298	EPI2864291	EPI2864297	EPI2864293	EPI2864292
A/Yunnan-Linxiang/11038/2017	EPI_ISL_18681136	EPI2864303	EPI2864304	EPI2864302	EPI2864306	EPI2864299	EPI2864305	EPI2864301	EPI2864300
A/Yunnan-Longyang/11032/2017	EPI_ISL_18681137	EPI2864311	EPI2864312	EPI2864310	EPI2864314	EPI2864307	EPI2864313	EPI2864309	EPI2864308
A/Yunnan-Linxiang/1656/2018	EPI_ISL_18681138	EPI2864319	EPI2864320	EPI2864318	EPI2864322	EPI2864315	EPI2864321	EPI2864317	EPI2864316
A/Yunnan-Linxiang/14/2018	EPI_ISL_18681139	EPI2864327	EPI2864328	EPI2864326	EPI2864330	EPI2864323	EPI2864329	EPI2864325	EPI2864324
A/Yunnan-Linxiang/123/2018	EPI_ISL_18681140	EPI2864335	EPI2864336	EPI2864334	EPI2864338	EPI2864331	EPI2864337	EPI2864333	EPI2864332
A/Yunnan-Linxiang/1660/2018	EPI_ISL_18681141	EPI2864343	EPI2864344	EPI2864342	EPI2864346	EPI2864339	EPI2864345	EPI2864341	EPI2864340
A/Yunnan-Linxiang/114/2018	EPI_ISL_18681142	EPI2864351	EPI2864352	EPI2864350	EPI2864354	EPI2864347	EPI2864353	EPI2864349	EPI2864348
A/Yunnan-Jinghong/176/2018	EPI_ISL_18681143	EPI2864359	EPI2864360	EPI2864358	EPI2864362	EPI2864355	EPI2864361	EPI2864357	EPI2864356
A/Yunnan-Gucheng/1863/2018	EPI_ISL_18681144	EPI2864367	EPI2864368	EPI2864366	EPI2864370	EPI2864363	EPI2864369	EPI2864365	EPI2864364
A/Yunnan-Linxiang/15/2018	EPI_ISL_18681145	EPI2864375	EPI2864376	EPI2864374	EPI2864378	EPI2864371	EPI2864377	EPI2864373	EPI2864372
A/Yunnan-Linxiang/16/2018	EPI_ISL_18681146	EPI2864383	EPI2864384	EPI2864382	EPI2864386	EPI2864379	EPI2864385	EPI2864381	EPI2864380
A/Yunnan-Ruili/3157/2018	EPI_ISL_18681147	EPI2864391	EPI2864392	EPI2864390	EPI2864394	EPI2864387	EPI2864393	EPI2864389	EPI2864388
A/Yunnan-Linxiang/1594/2018	EPI_ISL_18681148	EPI2864399	EPI2864400	EPI2864398	EPI2864402	EPI2864395	EPI2864401	EPI2864397	EPI2864396
A/Yunnan-Linxiang/1673/2018	EPI_ISL_18681149	EPI2864407	EPI2864408	EPI2864406	EPI2864410	EPI2864403	EPI2864409	EPI2864405	EPI2864404
A/Yunnan-Linxiang/1273/2018	EPI_ISL_18681150	EPI2864415	EPI2864416	EPI2864414	EPI2864418	EPI2864411	EPI2864417	EPI2864413	EPI2864412
A/Yunnan-Linxiang/120/2018	EPI_ISL_18681151	EPI2864423	EPI2864424	EPI2864422	EPI2864426	EPI2864419	EPI2864425	EPI2864421	EPI2864420

A/Yunnan-Zhaoyang/579/2018	EPI_ISL_18681152	EPI2864431	EPI2864432	EPI2864430	EPI2864434	EPI2864427	EPI2864433	EPI2864429	EPI2864428
A/Yunnan-Qilin/1183/2018	EPI_ISL_18681153	EPI2864439	EPI2864440	EPI2864438	EPI2864442	EPI2864435	EPI2864441	EPI2864437	EPI2864436
A/Yunnan-Mengzi/1920/2018	EPI_ISL_18681154	EPI2864447	EPI2864448	EPI2864446	EPI2864450	EPI2864443	EPI2864449	EPI2864445	EPI2864444
A/Yunnan-Xianggelila/1923/2018	EPI_ISL_18681155	EPI2864455	EPI2864456	EPI2864454	EPI2864458	EPI2864451	EPI2864457	EPI2864453	EPI2864452
A/Yunnan-Mengzi/1162/2019	EPI_ISL_18681156	EPI2864463	EPI2864464	EPI2864462	EPI2864466	EPI2864459	EPI2864465	EPI2864461	EPI2864460
A/Yunnan-Anning/1115/2019	EPI_ISL_18681157	EPI2864471	EPI2864472	EPI2864470	EPI2864474	EPI2864467	EPI2864473	EPI2864469	EPI2864468
A/Yunnan-Zhaoyang/1166/2019	EPI_ISL_18681158	EPI2864479	EPI2864480	EPI2864478	EPI2864482	EPI2864475	EPI2864481	EPI2864477	EPI2864476
A/Yunnan-Xianggelila/186/2019	EPI_ISL_18681159	EPI2864487	EPI2864488	EPI2864486	EPI2864490	EPI2864483	EPI2864489	EPI2864485	EPI2864484
A/Yunnan-Gucheng/1324/2019	EPI_ISL_18681160	EPI2864495	EPI2864496	EPI2864494	EPI2864498	EPI2864491	EPI2864497	EPI2864493	EPI2864492
A/Yunnan-Zhaoyang/1262/2019	EPI_ISL_18681161	EPI2864503	EPI2864504	EPI2864502	EPI2864506	EPI2864499	EPI2864505	EPI2864501	EPI2864500
A/Yunnan-Chuxiong/11474/2019	EPI_ISL_18681162	EPI2864511	EPI2864512	EPI2864510	EPI2864514	EPI2864507	EPI2864513	EPI2864509	EPI2864508
A/Yunnan-Dali/1618/2019	EPI_ISL_18681163	EPI2864519	EPI2864520	EPI2864518	EPI2864522	EPI2864515	EPI2864521	EPI2864517	EPI2864516
A/Yunnan-Guandu/313/2019	EPI_ISL_18681164	EPI2864527	EPI2864528	EPI2864526	EPI2864530	EPI2864523	EPI2864529	EPI2864525	EPI2864524
A/Yunnan-Wuhua/1670/2019	EPI_ISL_18681165	EPI2864535	EPI2864536	EPI2864534	EPI2864538	EPI2864531	EPI2864537	EPI2864533	EPI2864532
A/Yunnan-Lufeng/32/2019	EPI_ISL_18681166	EPI2864543	EPI2864544	EPI2864542	EPI2864546	EPI2864539	EPI2864545	EPI2864541	EPI2864540
A/Yunnan-Lushui/1359/2019	EPI_ISL_18681167	EPI2864551	EPI2864552	EPI2864550	EPI2864554	EPI2864547	EPI2864553	EPI2864549	EPI2864548
A/Yunnan-Wuhua/158/2019	EPI_ISL_18681358	EPI2866063	EPI2866064	EPI2866062	EPI2866066	EPI2866059	EPI2866065	EPI2866061	EPI2866060
A/Yunnan-Linxiang/1557/2019	EPI_ISL_18681168	EPI2864559	EPI2864560	EPI2864558	EPI2864562	EPI2864555	EPI2864561	EPI2864557	EPI2864556
A/Yunnan-Linxiang/1636/2019	EPI_ISL_18681169	EPI2864567	EPI2864568	EPI2864566	EPI2864570	EPI2864563	EPI2864569	EPI2864565	EPI2864564
A/Yunnan-Lushui/1447/2019	EPI_ISL_18681170	EPI2864575	EPI2864576	EPI2864574	EPI2864578	EPI2864571	EPI2864577	EPI2864573	EPI2864572
A/Yunnan-Yiliang/313/2019	EPI_ISL_18681171	EPI2864583	EPI2864584	EPI2864582	EPI2864586	EPI2864579	EPI2864585	EPI2864581	EPI2864580
A/Yunnan-Yongshan/32/2019	EPI_ISL_18681172	EPI2864591	EPI2864592	EPI2864590	EPI2864594	EPI2864587	EPI2864593	EPI2864589	EPI2864588
A/Yunnan-Zhaoyang/11121/2019	EPI_ISL_18681173	EPI2864599	EPI2864600	EPI2864598	EPI2864602	EPI2864595	EPI2864601	EPI2864597	EPI2864596
A/Yunnan-Anning/1199/2019	EPI_ISL_18681174	EPI2864607	EPI2864608	EPI2864606	EPI2864610	EPI2864603	EPI2864609	EPI2864605	EPI2864604
A/Yunnan-Qilin/1453/2019	EPI_ISL_18681175	EPI2864615	EPI2864616	EPI2864614	EPI2864618	EPI2864611	EPI2864617	EPI2864613	EPI2864612
A/Yunnan-Xiangyun/36/2019	EPI_ISL_18681176	EPI2864623	EPI2864624	EPI2864622	EPI2864626	EPI2864619	EPI2864625	EPI2864621	EPI2864620

A/Yunnan-Wenshan/1961/2019	EPI_ISL_18681177	EPI2864631	EPI2864632	EPI2864630	EPI2864634	EPI2864627	EPI2864633	EPI2864629	EPI2864628
A/Yunnan-Xishan/1290/2019	EPI_ISL_18681178	EPI2864639	EPI2864640	EPI2864638	EPI2864642	EPI2864635	EPI2864641	EPI2864637	EPI2864636
A/Yunnan-Gejiu/321/2019	EPI_ISL_18681179	EPI2864647	EPI2864648	EPI2864646	EPI2864650	EPI2864643	EPI2864649	EPI2864645	EPI2864644
A/Yunnan-Lushui/312/2019	EPI_ISL_18681180	EPI2864655	EPI2864656	EPI2864654	EPI2864658	EPI2864651	EPI2864657	EPI2864653	EPI2864652
A/Yunnan-Lushui/1379/2019	EPI_ISL_18681181	EPI2864663	EPI2864664	EPI2864662	EPI2864666	EPI2864659	EPI2864665	EPI2864661	EPI2864660
A/Yunnan-Dali/1600/2019	EPI_ISL_18681182	EPI2864671	EPI2864672	EPI2864670	EPI2864674	EPI2864667	EPI2864673	EPI2864669	EPI2864668
A/Yunnan-Lushui/329/2019	EPI_ISL_18681183	EPI2864679	EPI2864680	EPI2864678	EPI2864682	EPI2864675	EPI2864681	EPI2864677	EPI2864676
A/Yunnan/354/2019	EPI_ISL_18681184	EPI2864687	EPI2864688	EPI2864686	EPI2864690	EPI2864683	EPI2864689	EPI2864685	EPI2864684
A/Yunnan-Jinning/331/2019	EPI_ISL_18681185	EPI2864695	EPI2864696	EPI2864694	EPI2864698	EPI2864691	EPI2864697	EPI2864693	EPI2864692
A/Yunnan-Jinning/338/2019	EPI_ISL_18681186	EPI2864703	EPI2864704	EPI2864702	EPI2864706	EPI2864699	EPI2864705	EPI2864701	EPI2864700
A/Yunnan-Jinning/340/2019	EPI_ISL_18681187	EPI2864711	EPI2864712	EPI2864710	EPI2864714	EPI2864707	EPI2864713	EPI2864709	EPI2864708
A/Yunnan-Jinning/323/2019	EPI_ISL_18681188	EPI2864719	EPI2864720	EPI2864718	EPI2864722	EPI2864715	EPI2864721	EPI2864717	EPI2864716
A/Yunnan-Zhaoyang/11086/2019	EPI_ISL_18681189	EPI2864727	EPI2864728	EPI2864726	EPI2864730	EPI2864723	EPI2864729	EPI2864725	EPI2864724
A/Yunnan-Zhaoyang/11122/2019	EPI_ISL_18681190	EPI2864735	EPI2864736	EPI2864734	EPI2864738	EPI2864731	EPI2864737	EPI2864733	EPI2864732
A/Yunnan-Yiliang/35/2019	EPI_ISL_18681191	EPI2864743	EPI2864744	EPI2864742	EPI2864746	EPI2864739	EPI2864745	EPI2864741	EPI2864740
A/Yunnan-Eshan/329/2019	EPI_ISL_18681192	EPI2864751	EPI2864752	EPI2864750	EPI2864754	EPI2864747	EPI2864753	EPI2864749	EPI2864748
A/Yunnan-Jinghong/11245/2019	EPI_ISL_18681193	EPI2864759	EPI2864760	EPI2864758	EPI2864762	EPI2864755	EPI2864761	EPI2864757	EPI2864756
A/Yunnan-Jinghong/11454/2019	EPI_ISL_18681194	EPI2864767	EPI2864768	EPI2864766	EPI2864770	EPI2864763	EPI2864769	EPI2864765	EPI2864764
A/Yunnan-Jinghong/11351/2019	EPI_ISL_18681195	EPI2864775	EPI2864776	EPI2864774	EPI2864778	EPI2864771	EPI2864777	EPI2864773	EPI2864772
A/Yunnan-Jinghong/11342/2019	EPI_ISL_18681196	EPI2864783	EPI2864784	EPI2864782	EPI2864786	EPI2864779	EPI2864785	EPI2864781	EPI2864780
A/Yunnan-Mangshi/331/2019	EPI_ISL_18681197	EPI2864791	EPI2864792	EPI2864790	EPI2864794	EPI2864787	EPI2864793	EPI2864789	EPI2864788
A/Yunnan-Linxiang/1756/2019	EPI_ISL_18681198	EPI2864799	EPI2864800	EPI2864798	EPI2864802	EPI2864795	EPI2864801	EPI2864797	EPI2864796
A/Yunnan-Mangshi/1537/2019	EPI_ISL_18681199	EPI2864807	EPI2864808	EPI2864806	EPI2864810	EPI2864803	EPI2864809	EPI2864805	EPI2864804
A/Yunnan-Mangshi/1597/2019	EPI_ISL_18681200	EPI2864815	EPI2864816	EPI2864814	EPI2864818	EPI2864811	EPI2864817	EPI2864813	EPI2864812
A/Yunnan-Jinghong/11371/2019	EPI_ISL_18681201	EPI2864823	EPI2864824	EPI2864822	EPI2864826	EPI2864819	EPI2864825	EPI2864821	EPI2864820
A/Yunnan-Hongta/1689/2019	EPI_ISL_18681202	EPI2864831	EPI2864832	EPI2864830	EPI2864834	EPI2864827	EPI2864833	EPI2864829	EPI2864828

A/Yunnan-Guandu/1832/2019	EPI_ISL_18681203	EPI2864839	EPI2864840	EPI2864838	EPI2864842	EPI2864835	EPI2864841	EPI2864837	EPI2864836
A/Yunnan-Dali/1614/2019	EPI_ISL_18681204	EPI2864847	EPI2864848	EPI2864846	EPI2864850	EPI2864843	EPI2864849	EPI2864845	EPI2864844
A/Yunnan-Guandu/32/2019	EPI_ISL_18681205	EPI2864855	EPI2864856	EPI2864854	EPI2864858	EPI2864851	EPI2864857	EPI2864853	EPI2864852
A/Yunnan-Guandu/1898/2019	EPI_ISL_18681206	EPI2864863	EPI2864864	EPI2864862	EPI2864866	EPI2864859	EPI2864865	EPI2864861	EPI2864860
A/Yunnan-Mile/312/2019	EPI_ISL_18681207	EPI2864871	EPI2864872	EPI2864870	EPI2864874	EPI2864867	EPI2864873	EPI2864869	EPI2864868
A/Yunnan-Gejiu/365/2019	EPI_ISL_18681208	EPI2864879	EPI2864880	EPI2864878	EPI2864882	EPI2864875	EPI2864881	EPI2864877	EPI2864876
A/Yunnan/11056/2019	EPI_ISL_18681209	EPI2864887	EPI2864888	EPI2864886	EPI2864890	EPI2864883	EPI2864889	EPI2864885	EPI2864884
A/Yunnan/11102/2019	EPI_ISL_18681210	EPI2864895	EPI2864896	EPI2864894	EPI2864898	EPI2864891	EPI2864897	EPI2864893	EPI2864892
A/Yunnan/11108/2019	EPI_ISL_18681211	EPI2864903	EPI2864904	EPI2864902	EPI2864906	EPI2864899	EPI2864905	EPI2864901	EPI2864900
A/Yunnan-Lushui/11033/2019	EPI_ISL_18681212	EPI2864911	EPI2864912	EPI2864910	EPI2864914	EPI2864907	EPI2864913	EPI2864909	EPI2864908
A/Yunnan-Simao/11541/2019	EPI_ISL_18681213	EPI2864919	EPI2864920	EPI2864918	EPI2864922	EPI2864915	EPI2864921	EPI2864917	EPI2864916
A/Yunnan-Lushui/1999/2019	EPI_ISL_18681214	EPI2864927	EPI2864928	EPI2864926	EPI2864930	EPI2864923	EPI2864929	EPI2864925	EPI2864924
A/Yunnan-Lushui/11012/2019	EPI_ISL_18681215	EPI2864935	EPI2864936	EPI2864934	EPI2864938	EPI2864931	EPI2864937	EPI2864933	EPI2864932
A/Yunnan-Lushui/1989/2019	EPI_ISL_18681216	EPI2864943	EPI2864944	EPI2864942	EPI2864946	EPI2864939	EPI2864945	EPI2864941	EPI2864940
A/Yunnan-Lushui/11043/2019	EPI_ISL_18681217	EPI2864951	EPI2864952	EPI2864950	EPI2864954	EPI2864947	EPI2864953	EPI2864949	EPI2864948
A/Yunnan-Jinghong/11305/2019	EPI_ISL_18681218	EPI2864959	EPI2864960	EPI2864958	EPI2864962	EPI2864955	EPI2864961	EPI2864957	EPI2864956
A/Yunnan-Zhaoyang/1387/2019	EPI_ISL_18681219	EPI2864967	EPI2864968	EPI2864966	EPI2864970	EPI2864963	EPI2864969	EPI2864965	EPI2864964
A/Yunnan-Luoping/314/2019	EPI_ISL_18681220	EPI2864975	EPI2864976	EPI2864974	EPI2864978	EPI2864971	EPI2864977	EPI2864973	EPI2864972
A/Yunnan-Fumin/314/2019	EPI_ISL_18681221	EPI2864983	EPI2864984	EPI2864982	EPI2864986	EPI2864979	EPI2864985	EPI2864981	EPI2864980
A/Yunnan-Mengzi/1910/2019	EPI_ISL_18681222	EPI2864991	EPI2864992	EPI2864990	EPI2864994	EPI2864987	EPI2864993	EPI2864989	EPI2864988
A/Yunnan-Simao/12116/2019	EPI_ISL_18681223	EPI2864999	EPI2865000	EPI2864998	EPI2865002	EPI2864995	EPI2865001	EPI2864997	EPI2864996
A/Yunnan-Simao/12179/2019	EPI_ISL_18681224	EPI2865007	EPI2865008	EPI2865006	EPI2865010	EPI2865003	EPI2865009	EPI2865005	EPI2865004
A/Yunnan-Yingjiang/363/2019	EPI_ISL_18681225	EPI2865015	EPI2865016	EPI2865014	EPI2865018	EPI2865011	EPI2865017	EPI2865013	EPI2865012
A/Yunnan-Shiping/34/2019	EPI_ISL_18681226	EPI2865023	EPI2865024	EPI2865022	EPI2865026	EPI2865019	EPI2865025	EPI2865021	EPI2865020
A/Yunnan-Fumin/312/2019	EPI_ISL_18681227	EPI2865031	EPI2865032	EPI2865030	EPI2865034	EPI2865027	EPI2865033	EPI2865029	EPI2865028
A/Yunnan-Gejiu/325/2019	EPI_ISL_18681228	EPI2865039	EPI2865040	EPI2865038	EPI2865042	EPI2865035	EPI2865041	EPI2865037	EPI2865036

A/Yunnan-Mengzi/1856/2019	EPI_ISL_18681229	EPI2865047	EPI2865048	EPI2865046	EPI2865050	EPI2865043	EPI2865049	EPI2865045	EPI2865044
A/Yunnan-Mengzi/1882/2019	EPI_ISL_18681230	EPI2865055	EPI2865056	EPI2865054	EPI2865058	EPI2865051	EPI2865057	EPI2865053	EPI2865052
A/Yunnan-Wenshan/11039/2019	EPI_ISL_18681231	EPI2865063	EPI2865064	EPI2865062	EPI2865066	EPI2865059	EPI2865065	EPI2865061	EPI2865060
A/Yunnan-Mengzi/1764/2019	EPI_ISL_18681232	EPI2865071	EPI2865072	EPI2865070	EPI2865074	EPI2865067	EPI2865073	EPI2865069	EPI2865068
A/Yunnan-Mengzi/1866/2019	EPI_ISL_18681233	EPI2865079	EPI2865080	EPI2865078	EPI2865082	EPI2865075	EPI2865081	EPI2865077	EPI2865076
A/Yunnan-Gucheng/138/2020	EPI_ISL_18681234	EPI2865087	EPI2865088	EPI2865086	EPI2865090	EPI2865083	EPI2865089	EPI2865085	EPI2865084
A/Yunnan-Longyang/180/2020	EPI_ISL_18681235	EPI2865095	EPI2865096	EPI2865094	EPI2865098	EPI2865091	EPI2865097	EPI2865093	EPI2865092
A/Yunnan-Linxiang/1657/2020	EPI_ISL_18681236	EPI2865103	EPI2865104	EPI2865102	EPI2865106	EPI2865099	EPI2865105	EPI2865101	EPI2865100
A/Yunnan-Linxiang/1668/2020	EPI_ISL_18681237	EPI2865111	EPI2865112	EPI2865110	EPI2865114	EPI2865107	EPI2865113	EPI2865109	EPI2865108
A/Yunnan-Linxiang/1480/2020	EPI_ISL_18681238	EPI2865119	EPI2865120	EPI2865118	EPI2865122	EPI2865115	EPI2865121	EPI2865117	EPI2865116
A/Yunnan-Linxiang/1677/2020	EPI_ISL_18681239	EPI2865127	EPI2865128	EPI2865126	EPI2865130	EPI2865123	EPI2865129	EPI2865125	EPI2865124
A/Yunnan-Chuxiong/157/2020	EPI_ISL_18681240	EPI2865135	EPI2865136	EPI2865134	EPI2865138	EPI2865131	EPI2865137	EPI2865133	EPI2865132
A/Yunnan-Wenshan/1599/2020	EPI_ISL_18681241	EPI2865143	EPI2865144	EPI2865142	EPI2865146	EPI2865139	EPI2865145	EPI2865141	EPI2865140
A/Yunnan-Jinghong/122/2020	EPI_ISL_18681242	EPI2865151	EPI2865152	EPI2865150	EPI2865154	EPI2865147	EPI2865153	EPI2865149	EPI2865148
A/Yunnan-Jinghong/150/2020	EPI_ISL_18681243	EPI2865159	EPI2865160	EPI2865158	EPI2865162	EPI2865155	EPI2865161	EPI2865157	EPI2865156
A/Yunnan-Linxiang/1650/2020	EPI_ISL_18681244	EPI2865167	EPI2865168	EPI2865166	EPI2865170	EPI2865163	EPI2865169	EPI2865165	EPI2865164
A/Yunnan-Linxiang/1670/2020	EPI_ISL_18681245	EPI2865175	EPI2865176	EPI2865174	EPI2865178	EPI2865171	EPI2865177	EPI2865173	EPI2865172
A/Yunnan-Wenshan/1487/2020	EPI_ISL_18681246	EPI2865183	EPI2865184	EPI2865182	EPI2865186	EPI2865179	EPI2865185	EPI2865181	EPI2865180
A/Yunnan-Guandu/18/2020	EPI_ISL_18681247	EPI2865191	EPI2865192	EPI2865190	EPI2865194	EPI2865187	EPI2865193	EPI2865189	EPI2865188
A/Yunnan-Wenshan/1485/2020	EPI_ISL_18681248	EPI2865199	EPI2865200	EPI2865198	EPI2865202	EPI2865195	EPI2865201	EPI2865197	EPI2865196
A/Yunnan-Gucheng/1268/2020	EPI_ISL_18681249	EPI2865207	EPI2865208	EPI2865206	EPI2865210	EPI2865203	EPI2865209	EPI2865205	EPI2865204
A/Yunnan-Wenshan/1424/2020	EPI_ISL_18681250	EPI2865215	EPI2865216	EPI2865214	EPI2865218	EPI2865211	EPI2865217	EPI2865213	EPI2865212
A/Yunnan-Jinghong/11/2020	EPI_ISL_18681251	EPI2865223	EPI2865224	EPI2865222	EPI2865226	EPI2865219	EPI2865225	EPI2865221	EPI2865220
A/Yunnan-Wenshan/1435/2020	EPI_ISL_18681252	EPI2865231	EPI2865232	EPI2865230	EPI2865234	EPI2865227	EPI2865233	EPI2865229	EPI2865228
A/Yunnan-Wenshan/1476/2020	EPI_ISL_18681253	EPI2865239	EPI2865240	EPI2865238	EPI2865242	EPI2865235	EPI2865241	EPI2865237	EPI2865236
A/Yunnan-Wenshan/1481/2020	EPI_ISL_18681254	EPI2865247	EPI2865248	EPI2865246	EPI2865250	EPI2865243	EPI2865249	EPI2865245	EPI2865244

A/Yunnan-Wenshan/1606/2020	EPI_ISL_18681255	EPI2865255	EPI2865256	EPI2865254	EPI2865258	EPI2865251	EPI2865257	EPI2865253	EPI2865252
A/Yunnan-Wenshan/1594/2020	EPI_ISL_18681256	EPI2865263	EPI2865264	EPI2865262	EPI2865266	EPI2865259	EPI2865265	EPI2865261	EPI2865260
A/Yunnan-Wenshan/1593/2020	EPI_ISL_18681257	EPI2865271	EPI2865272	EPI2865270	EPI2865274	EPI2865267	EPI2865273	EPI2865269	EPI2865268
A/Yunnan-Wenshan/1592/2020	EPI_ISL_18681258	EPI2865279	EPI2865280	EPI2865278	EPI2865282	EPI2865275	EPI2865281	EPI2865277	EPI2865276
A/Yunnan-Wenshan/1483/2020	EPI_ISL_18681259	EPI2865287	EPI2865288	EPI2865286	EPI2865290	EPI2865283	EPI2865289	EPI2865285	EPI2865284
A/Yunnan-Wenshan/1489/2020	EPI_ISL_18681260	EPI2865295	EPI2865296	EPI2865294	EPI2865298	EPI2865291	EPI2865297	EPI2865293	EPI2865292
A/Yunnan-Jinghong/168/2020	EPI_ISL_18681261	EPI2865303	EPI2865304	EPI2865302	EPI2865306	EPI2865299	EPI2865305	EPI2865301	EPI2865300
A/Yunnan-Qilin/11/2020	EPI_ISL_18681262	EPI2865311	EPI2865312	EPI2865310	EPI2865314	EPI2865307	EPI2865313	EPI2865309	EPI2865308
A/Yunnan-Longyang/164/2020	EPI_ISL_18681263	EPI2865319	EPI2865320	EPI2865318	EPI2865322	EPI2865315	EPI2865321	EPI2865317	EPI2865316
A/Yunnan-Linxiang/130/2021	EPI_ISL_18681264	EPI2865327	EPI2865328	EPI2865326	EPI2865330	EPI2865323	EPI2865329	EPI2865325	EPI2865324
A/Yunnan-Linxiang/132/2021	EPI_ISL_18681265	EPI2865335	EPI2865336	EPI2865334	EPI2865338	EPI2865331	EPI2865337	EPI2865333	EPI2865332
A/Yunnan-Wenshan/1209/2021	EPI_ISL_18681266	EPI2865343	EPI2865344	EPI2865342	EPI2865346	EPI2865339	EPI2865345	EPI2865341	EPI2865340
A/Yunnan-Wenshan/1199/2021	EPI_ISL_18681267	EPI2865351	EPI2865352	EPI2865350	EPI2865354	EPI2865347	EPI2865353	EPI2865349	EPI2865348
A/Yunnan-Wenshan/1183/2021	EPI_ISL_18681268	EPI2865359	EPI2865360	EPI2865358	EPI2865362	EPI2865355	EPI2865361	EPI2865357	EPI2865356
A/Yunnan-Wenshan/1193/2021	EPI_ISL_18681269	EPI2865367	EPI2865368	EPI2865366	EPI2865370	EPI2865363	EPI2865369	EPI2865365	EPI2865364
A/Yunnan-Wenshan/1213/2021	EPI_ISL_18681270	EPI2865375	EPI2865376	EPI2865374	EPI2865378	EPI2865371	EPI2865377	EPI2865373	EPI2865372
A/Yunnan-Wenshan/1218/2021	EPI_ISL_18681271	EPI2865383	EPI2865384	EPI2865382	EPI2865386	EPI2865379	EPI2865385	EPI2865381	EPI2865380
A/Yunnan-Chuxiong/1459/2022	EPI_ISL_18681272	EPI2865391	EPI2865392	EPI2865390	EPI2865394	EPI2865387	EPI2865393	EPI2865389	EPI2865388
A/Yunnan-Chuxiong/1471/2022	EPI_ISL_18681273	EPI2865399	EPI2865400	EPI2865398	EPI2865402	EPI2865395	EPI2865401	EPI2865397	EPI2865396
A/Yunnan-Chuxiong/317/2022	EPI_ISL_18681274	EPI2865407	EPI2865408	EPI2865406	EPI2865410	EPI2865403	EPI2865409	EPI2865405	EPI2865404
A/Yunnan-Chuxiong/1499/2022	EPI_ISL_18681275	EPI2865415	EPI2865416	EPI2865414	EPI2865418	EPI2865411	EPI2865417	EPI2865413	EPI2865412
A/Yunnan-Chuxiong/351/2022	EPI_ISL_18681276	EPI2865423	EPI2865424	EPI2865422	EPI2865426	EPI2865419	EPI2865425	EPI2865421	EPI2865420
A/Yunnan-Chuxiong/349/2022	EPI_ISL_18681277	EPI2865431	EPI2865432	EPI2865430	EPI2865434	EPI2865427	EPI2865433	EPI2865429	EPI2865428
A/Yunnan-Chuxiong/348/2022	EPI_ISL_18681278	EPI2865439	EPI2865440	EPI2865438	EPI2865442	EPI2865435	EPI2865441	EPI2865437	EPI2865436
A/Yunnan-Wenshan/1576/2022	EPI_ISL_18681279	EPI2865447	EPI2865448	EPI2865446	EPI2865450	EPI2865443	EPI2865449	EPI2865445	EPI2865444
A/Yunnan-Wenshan/1591/2022	EPI_ISL_18681280	EPI2865455	EPI2865456	EPI2865454	EPI2865458	EPI2865451	EPI2865457	EPI2865453	EPI2865452

A/Yunnan-Anning/11018/2022	EPI_ISL_18681281	EPI2865463	EPI2865464	EPI2865462	EPI2865466	EPI2865459	EPI2865465	EPI2865461	EPI2865460
A/Yunnan-Anning/11119/2022	EPI_ISL_18681282	EPI2865471	EPI2865472	EPI2865470	EPI2865474	EPI2865467	EPI2865473	EPI2865469	EPI2865468
A/Yunnan-Anning/11150/2022	EPI_ISL_18681283	EPI2865479	EPI2865480	EPI2865478	EPI2865482	EPI2865475	EPI2865481	EPI2865477	EPI2865476
A/Yunnan-Anning/11181/2022	EPI_ISL_18681284	EPI2865487	EPI2865488	EPI2865486	EPI2865490	EPI2865483	EPI2865489	EPI2865485	EPI2865484
A/Yunnan-Anning/11216/2022	EPI_ISL_18681285	EPI2865495	EPI2865496	EPI2865494	EPI2865498	EPI2865491	EPI2865497	EPI2865493	EPI2865492
A/Yunnan-Anning/11171/2022	EPI_ISL_18681286	EPI2865503	EPI2865504	EPI2865502	EPI2865506	EPI2865499	EPI2865505	EPI2865501	EPI2865500
A/Yunnan-Mangshi/11029/2022	EPI_ISL_18681287	EPI2865511	EPI2865512	EPI2865510	EPI2865514	EPI2865507	EPI2865513	EPI2865509	EPI2865508
A/Yunnan-Mengzi/1386/2022	EPI_ISL_18681288	EPI2865519	EPI2865520	EPI2865518	EPI2865522	EPI2865515	EPI2865521	EPI2865517	EPI2865516
A/Yunnan-Anning/11177/2022	EPI_ISL_18681289	EPI2865527	EPI2865528	EPI2865526	EPI2865530	EPI2865523	EPI2865529	EPI2865525	EPI2865524
A/Yunnan-Anning/1778/2022	EPI_ISL_18681290	EPI2865535	EPI2865536	EPI2865534	EPI2865538	EPI2865531	EPI2865537	EPI2865533	EPI2865532
A/Yunnan-Anning/1813/2022	EPI_ISL_18681291	EPI2865543	EPI2865544	EPI2865542	EPI2865546	EPI2865539	EPI2865545	EPI2865541	EPI2865540
A/Yunnan-Mangshi/1464/2022	EPI_ISL_18681292	EPI2865551	EPI2865552	EPI2865550	EPI2865554	EPI2865547	EPI2865553	EPI2865549	EPI2865548
A/Yunnan-Mangshi/1610/2022	EPI_ISL_18681293	EPI2865559	EPI2865560	EPI2865558	EPI2865562	EPI2865555	EPI2865561	EPI2865557	EPI2865556
A/Yunnan-Mangshi/1667/2022	EPI_ISL_18681294	EPI2865567	EPI2865568	EPI2865566	EPI2865570	EPI2865563	EPI2865569	EPI2865565	EPI2865564
A/Yunnan-Mangshi/1888/2022	EPI_ISL_18681295	EPI2865575	EPI2865576	EPI2865574	EPI2865578	EPI2865571	EPI2865577	EPI2865573	EPI2865572
A/Yunnan-Mangshi/11006/2022	EPI_ISL_18681296	EPI2865583	EPI2865584	EPI2865582	EPI2865586	EPI2865579	EPI2865585	EPI2865581	EPI2865580
A/Yunnan-Mangshi/349/2022	EPI_ISL_18681297	EPI2865591	EPI2865592	EPI2865590	EPI2865594	EPI2865587	EPI2865593	EPI2865589	EPI2865588
A/Yunnan-Mangshi/1907/2022	EPI_ISL_18681298	EPI2865599	EPI2865600	EPI2865598	EPI2865602	EPI2865595	EPI2865601	EPI2865597	EPI2865596
A/Yunnan-Mangshi/1852/2022	EPI_ISL_18681299	EPI2865607	EPI2865608	EPI2865606	EPI2865610	EPI2865603	EPI2865609	EPI2865605	EPI2865604
A/Yunnan-Mangshi/1925/2022	EPI_ISL_18681300	EPI2865615	EPI2865616	EPI2865614	EPI2865618	EPI2865611	EPI2865617	EPI2865613	EPI2865612
A/Yunnan-Mangshi/1938/2022	EPI_ISL_18681301	EPI2865623	EPI2865624	EPI2865622	EPI2865626	EPI2865619	EPI2865625	EPI2865621	EPI2865620
A/Yunnan-Mangshi/1990/2022	EPI_ISL_18681302	EPI2865631	EPI2865632	EPI2865630	EPI2865634	EPI2865627	EPI2865633	EPI2865629	EPI2865628
A/Yunnan-Linxiang/1183/2022	EPI_ISL_18681303	EPI2865639	EPI2865640	EPI2865638	EPI2865642	EPI2865635	EPI2865641	EPI2865637	EPI2865636
A/Yunnan-Simao/1948/2022	EPI_ISL_18681304	EPI2865647	EPI2865648	EPI2865646	EPI2865650	EPI2865643	EPI2865649	EPI2865645	EPI2865644
A/Yunnan-Funing/310/2022	EPI_ISL_18681305	EPI2865655	EPI2865656	EPI2865654	EPI2865658	EPI2865651	EPI2865657	EPI2865653	EPI2865652
A/Yunnan-Hongta/11014/2022	EPI_ISL_18681306	EPI2865663	EPI2865664	EPI2865662	EPI2865666	EPI2865659	EPI2865665	EPI2865661	EPI2865660

A/Yunnan-Zhenxiong/533/2022	EPI_ISL_18681307	EPI2865671	EPI2865672	EPI2865670	EPI2865674	EPI2865667	EPI2865673	EPI2865669	EPI2865668
A/Yunnan-Zhaoyang/1405/2022	EPI_ISL_18681308	EPI2865679	EPI2865680	EPI2865678	EPI2865682	EPI2865675	EPI2865681	EPI2865677	EPI2865676
A/Yunnan-Zhaoyang/1402/2022	EPI_ISL_18681309	EPI2865687	EPI2865688	EPI2865686	EPI2865690	EPI2865683	EPI2865689	EPI2865685	EPI2865684
A/Yunnan-Hongta/11154/2022	EPI_ISL_18681310	EPI2865695	EPI2865696	EPI2865694	EPI2865698	EPI2865691	EPI2865697	EPI2865693	EPI2865692
A/Yunnan-Hongta/11161/2022	EPI_ISL_18681311	EPI2865703	EPI2865704	EPI2865702	EPI2865706	EPI2865699	EPI2865705	EPI2865701	EPI2865700
A/Yunnan-Qilin/1421/2022	EPI_ISL_18681312	EPI2865711	EPI2865712	EPI2865710	EPI2865714	EPI2865707	EPI2865713	EPI2865709	EPI2865708
A/Yunnan-Qilin/1487/2022	EPI_ISL_18681313	EPI2865719	EPI2865720	EPI2865718	EPI2865722	EPI2865715	EPI2865721	EPI2865717	EPI2865716
A/Yunnan-Qilin/1467/2022	EPI_ISL_18681314	EPI2865727	EPI2865728	EPI2865726	EPI2865730	EPI2865723	EPI2865729	EPI2865725	EPI2865724
A/Yunnan-Qilin/1475/2022	EPI_ISL_18681315	EPI2865735	EPI2865736	EPI2865734	EPI2865738	EPI2865731	EPI2865737	EPI2865733	EPI2865732
A/Yunnan-Zhenxiong/530/2022	EPI_ISL_18681316	EPI2865743	EPI2865744	EPI2865742	EPI2865746	EPI2865739	EPI2865745	EPI2865741	EPI2865740
A/Yunnan-Zhaoyang/1414/2022	EPI_ISL_18681317	EPI2865751	EPI2865752	EPI2865750	EPI2865754	EPI2865747	EPI2865753	EPI2865749	EPI2865748
A/Yunnan-Zhenxiong/531/2022	EPI_ISL_18681318	EPI2865759	EPI2865760	EPI2865758	EPI2865762	EPI2865755	EPI2865761	EPI2865757	EPI2865756
A/Yunnan-Qilin/1483/2022	EPI_ISL_18681319	EPI2865767	EPI2865768	EPI2865766	EPI2865770	EPI2865763	EPI2865769	EPI2865765	EPI2865764
A/Yunnan-Mengzi/1497/2022	EPI_ISL_18681320	EPI2865775	EPI2865776	EPI2865774	EPI2865778	EPI2865771	EPI2865777	EPI2865773	EPI2865772
A/Yunnan-Malipo/51/2022	EPI_ISL_18681321	EPI2865783	EPI2865784	EPI2865782	EPI2865786	EPI2865779	EPI2865785	EPI2865781	EPI2865780
A/Yunnan-Funing/314/2022	EPI_ISL_18681322	EPI2865791	EPI2865792	EPI2865790	EPI2865794	EPI2865787	EPI2865793	EPI2865789	EPI2865788
A/Yunnan-Hongta/11144/2022	EPI_ISL_18681323	EPI2865799	EPI2865800	EPI2865798	EPI2865802	EPI2865795	EPI2865801	EPI2865797	EPI2865796
A/Yunnan-Yingjiang/324/2022	EPI_ISL_18681324	EPI2865807	EPI2865808	EPI2865806	EPI2865810	EPI2865803	EPI2865809	EPI2865805	EPI2865804
A/Yunnan-Linxiang/1188/2022	EPI_ISL_18681325	EPI2865815	EPI2865816	EPI2865814	EPI2865818	EPI2865811	EPI2865817	EPI2865813	EPI2865812
A/Yunnan-Mangshi/1732/2022	EPI_ISL_18681326	EPI2865823	EPI2865824	EPI2865822	EPI2865826	EPI2865819	EPI2865825	EPI2865821	EPI2865820
A/Yunnan-Yingjiang/359/2022	EPI_ISL_18681327	EPI2865831	EPI2865832	EPI2865830	EPI2865834	EPI2865827	EPI2865833	EPI2865829	EPI2865828
A/Yunnan-Gejiu/345/2022	EPI_ISL_18681328	EPI2865839	EPI2865840	EPI2865838	EPI2865842	EPI2865835	EPI2865841	EPI2865837	EPI2865836
A/Yunnan-Gejiu/343/2022	EPI_ISL_18681329	EPI2865847	EPI2865848	EPI2865846	EPI2865850	EPI2865843	EPI2865849	EPI2865845	EPI2865844
A/Yunnan-Gejiu/335/2022	EPI_ISL_18681330	EPI2865855	EPI2865856	EPI2865854	EPI2865858	EPI2865851	EPI2865857	EPI2865853	EPI2865852
A/Yunnan-Hongta/11028/2022	EPI_ISL_18681331	EPI2865863	EPI2865864	EPI2865862	EPI2865866	EPI2865859	EPI2865865	EPI2865861	EPI2865860
A/Yunnan-Gejiu/329/2022	EPI_ISL_18681332	EPI2865871	EPI2865872	EPI2865870	EPI2865874	EPI2865867	EPI2865873	EPI2865869	EPI2865868

A/Yunnan-Linxiang/1212/2022	EPI_ISL_18681333	EPI2865879	EPI2865880	EPI2865878	EPI2865882	EPI2865875	EPI2865881	EPI2865877	EPI2865876
A/Yunnan-Mengzi/1377/2022	EPI_ISL_18681334	EPI2865887	EPI2865888	EPI2865886	EPI2865890	EPI2865883	EPI2865889	EPI2865885	EPI2865884
A/Yunnan-Linxiang/1206/2022	EPI_ISL_18681335	EPI2865895	EPI2865896	EPI2865894	EPI2865898	EPI2865891	EPI2865897	EPI2865893	EPI2865892
A/Yunnan-Linxiang/1216/2022	EPI_ISL_18681336	EPI2865903	EPI2865904	EPI2865902	EPI2865906	EPI2865899	EPI2865905	EPI2865901	EPI2865900
A/Yunnan-Jinping/399/2022	EPI_ISL_18681337	EPI2865911	EPI2865912	EPI2865910	EPI2865914	EPI2865907	EPI2865913	EPI2865909	EPI2865908
A/Yunnan-Malipo/37/2022	EPI_ISL_18681338	EPI2865919	EPI2865920	EPI2865918	EPI2865922	EPI2865915	EPI2865921	EPI2865917	EPI2865916
A/Yunnan-Xishan/11119/2022	EPI_ISL_18681339	EPI2865927	EPI2865928	EPI2865926	EPI2865930	EPI2865923	EPI2865929	EPI2865925	EPI2865924
A/Yunnan-Qilin/1582/2022	EPI_ISL_18681340	EPI2865935	EPI2865936	EPI2865934	EPI2865938	EPI2865931	EPI2865937	EPI2865933	EPI2865932
A/Yunnan-Qilin/1583/2022	EPI_ISL_18681341	EPI2865943	EPI2865944	EPI2865942	EPI2865946	EPI2865939	EPI2865945	EPI2865941	EPI2865940
A/Yunnan-Mengzi/1387/2022	EPI_ISL_18681342	EPI2865951	EPI2865952	EPI2865950	EPI2865954	EPI2865947	EPI2865953	EPI2865949	EPI2865948
A/Yunnan-Qilin/1959/2022	EPI_ISL_18681343	EPI2865959	EPI2865960	EPI2865958	EPI2865962	EPI2865955	EPI2865961	EPI2865957	EPI2865956
A/Yunnan-Hongta/11149/2022	EPI_ISL_18681344	EPI2865967	EPI2865968	EPI2865966	EPI2865970	EPI2865963	EPI2865969	EPI2865965	EPI2865964
A/Yunnan-Lushui/1989/2022	EPI_ISL_18681345	EPI2865975	EPI2865976	EPI2865974	EPI2865978	EPI2865971	EPI2865977	EPI2865973	EPI2865972
A/Yunnan-Hongta/11025/2022	EPI_ISL_18681346	EPI2865983	EPI2865984	EPI2865982	EPI2865986	EPI2865979	EPI2865985	EPI2865981	EPI2865980
A/Yunnan-Wenshan/1633/2022	EPI_ISL_18681347	EPI2865991	EPI2865992	EPI2865990	EPI2865994	EPI2865987	EPI2865993	EPI2865989	EPI2865988
A/Yunnan-Wenshan/1710/2022	EPI_ISL_18681348	EPI2865999	EPI2866000	EPI2865998	EPI2866002	EPI2865995	EPI2866001	EPI2865997	EPI2865996
A/Yunnan-Wuhua/1909/2022	EPI_ISL_18681349	EPI2866007	EPI2866008	EPI2866006	EPI2866010	EPI2866003	EPI2866009	EPI2866005	EPI2866004
A/Yunnan-Xianggelila/1928/2022	EPI_ISL_18681350	EPI2866015	EPI2866016	EPI2866014	EPI2866018	EPI2866011	EPI2866017	EPI2866013	EPI2866012
A/Yunnan-Xianggelila/1944/2022	EPI_ISL_18681351	EPI2866023	EPI2866024	EPI2866022	EPI2866026	EPI2866019	EPI2866025	EPI2866021	EPI2866020
A/Yunnan-Xianggelila/387/2022	EPI_ISL_18681352	EPI2866031	EPI2866032	EPI2866030	EPI2866034	EPI2866027	EPI2866033	EPI2866029	EPI2866028
A/Yunnan-Xianggelila/11226/2022	EPI_ISL_18681353	EPI2866039	EPI2866040	EPI2866038	EPI2866042	EPI2866035	EPI2866041	EPI2866037	EPI2866036
A/Yunnan-Xianggelila/11239/2022	EPI_ISL_18681354	EPI2866047	EPI2866048	EPI2866046	EPI2866050	EPI2866043	EPI2866049	EPI2866045	EPI2866044
A/Yunnan-Xianggelila/11216/2022	EPI_ISL_18681355	EPI2866055	EPI2866056	EPI2866054	EPI2866058	EPI2866051	EPI2866057	EPI2866053	EPI2866052
A/Texas/50/2012	EPI_ISL_16701302	EPI2318459	EPI2318460	EPI2318461	EPI2318462	EPI2318463	EPI2318464	EPI2318465	EPI2318466
A/Hong Kong/4801/2014	EPI_ISL_202569	EPI696096	EPI675796	EPI696095	EPI675798	EPI675793	EPI675797	EPI675795	EPI675794
A/Singapore/INFIMH-16-0019/2016	EPI_ISL_296168	EPI1164033	EPI1164034	EPI1164032	EPI1164036	EPI1164029	EPI1164035	EPI1164031	EPI1164030

A/Kansas/14/2017	EPI_ISL_298310	EPI1174040	EPI117404	EPI1174039	EPI1174043	EPI1174036	EPI1174042	EPI1174038	EPI1174037
A/Hong Kong/45/2019	EPI_ISL_347938	EPI1395763	EPI1397374	EPI1397373	EPI1397376	EPI1395762	EPI1397375	EPI1397372	EPI1397371
A/Cambodia/e0826360/2020	EPI_ISL_710475	EPI1841681	EPI1841682	EPI1841680	EPI1837753	EPI1841678	EPI1837752	EPI1837751	EPI1841679
A/Darwin/6/2021	EPI_ISL_5332906	EPI1922119	EPI1922120	EPI1922118	EPI1922122	EPI1922115	EPI1922121	EPI1922117	EPI1922116
A/California/18/2014	EPI_ISL_167918	EPI547787	EPI547788	EPI547786	EPI547790	EPI547783	EPI547789	EPI547785	EPI547784
A/Switzerland/9715293/2013		OQ350029	OQ350030	OQ350031	OQ350032	OQ350033	OQ350034	OQ350035	OQ350036
A/Newcastle/184/2017		MN569638	MN569637	MN569636	MN569631	MN569634	MN569633	MN569632	MN569635
A/New York/PV37/2017		MH798605	MH798604	MH798603	MH798598	MH798601	MH798600.	MH798599	MH798602
A/Switzerland/8060/2017		OQ350846	OQ350847	OQ350848	OQ350849	OQ350850	OQ350851	OQ350852	OQ350853
A/Thailand/B29296/2018		MN561350	MN561351	MN561352	MN561353	MN561354	MN561355	MN561356	MN561357
A/Victoria/829/2017		MN574190	MN574189	MN574188	MN574183	MN574186	MN574185	MN574184	MN574187
A/Newcastle/4/2018		MN569798	MN569797	MN569796	MN569791	MN569794	MN569793	MN569792	MN569795
A/Perth/352/2017		MN570222	MN570221	MN570220	MN570215	MN570218	MN570217	MN570216	MN570219
A/South Australia/149/2017		MN570550	MN570549	MN570548	MN570543	MN570546	MN570545	MN570544	MN570547
A/Thailand/B24666/2017		MN561330	MN561331	MN561332	MN561333	MN561334	MN561335	MN561336	MN561337
A/South Australia/34/2019	EPI_ISL_398939	EPI1631899	EPI1631900	EPI1631898	EPI1631902	EPI1631895	EPI1631901	EPI1631897	EPI1631896
A/North Dakota/38/2018		MK363363	MK265457	MK265458	MK265459	MK265460	MK265461	MK265462	MK265463
A/Minnesota/41/2019		MN372908	MN372909	MN372910	MN372911	MN372912	MN372913	MN372914	MN372915
A/Washington/47/2022		OQ234824	OQ234825	OQ234826	OQ234827	OQ234828	OQ234829	OQ234830	OQ234831

Abbreviations: PB2, polymerase basic 2; PB1, polymerase basic 1; PA, polymerase acid; HA, hemagglutinin; NP, nucleocapsid protein; NA, neuraminidase; M, matrix protein; NS, nonstructural protein.
