# Supplementary material for: Genomic Analyses Uncover Evolutionary Features of Influenza A/H3N2 Viruses in Yunnan Province, China, from 2017 to 2022
Source: Viruses. 2024 Jan 18;16(1):138. doi: 10.3390/v16010138 (PMC10820241; doi:10.3390/v16010138)
Supplement: Supplementary file 1 [file viruses-16-00138-s001.zip › Table S2.pdf]

Table S2. The number (%) of influenza strains isolated from samples of influenza-like cases in Yunnan Province, China, from 2017 to 2022.

Year	Number of samples tested	Number (%) of isolated strains			
		Type A		Type B	Total (each year)
		A/H3N2	A/H1N1		
2017	22721	865 (3.81)		475(2.10)	1340(5.90)
		366 (42.31)	499 (57.69)		
2018	22294	865 (3.88)		277(1.24)	1142(5.12)
		70 (8.09)	795 (91.91)		
2019	22700	1004 (4.42)		346(1.52)	1350(5.95)
		504 (50.20)	500 (49.80)		
2020	19780	191 (0.97)		45(0.23)	236(1.19)
		79 (41.36)	112 (58.64)		
2021	26292	9 (0.03)		303(1.15)	312(1.19)
		8 (88.89)	1 (11.11)		
2022	23209	345 (1.49)		366(1.58)	711(3.06)
		345 (100.00)	0 (0.00)		
Total (all years)	136996	3279 (2.39)		1812(1.32)	5091(3.72)
		1372(41.84)	1907(58.16)		
