# Supplementary material for: Genomic Analyses Uncover Evolutionary Features of Influenza A/H3N2 Viruses in Yunnan Province, China, from 2017 to 2022
Source: Viruses. 2024 Jan 18;16(1):138. doi: 10.3390/v16010138 (PMC10820241; doi:10.3390/v16010138)
Supplement: Supplementary file 1 [file viruses-16-00138-s001.zip › Table S3.pdf]

Table S3. Demographic characteristics of the cases infected with influenza viruses in Yunnan Province, China, from 2017 to 2022.

Classification	Number (%) of the cases infected with influenza viruses						
**Year (Total cases)**	2017 (1340)	2018 (1142)	2019 (1350)	2020 (236)	2021 (312)	2022 (711)	Total (5091)
**Sex**							
female	606 (45.22)	514 (45.01)	606 (44.89)	94 (39.83)	128 (41.03)	299 (42.05)	2247 (44.14)
male	734 (54.78)	628 (54.99)	744 (55.11)	142 (60.17)	184 (58.97)	412 (57.95)	2844 (55.86)
**Age group (years)**							
0～	408 (30.45)	320 (28.02)	343 (25.41)	104 (44.07)	29 (9.29)	151 (21.24)	1355 (26.62)
5～	437 (32.61)	372 (32.57)	618 (45.78)	45 (19.07)	156 (50)	234 (32.91)	1862 (36.57)
15～	151 (11.27)	157 (13.75)	179 (13.26)	31 (13.14)	70 (22.44)	176 (24.75)	764 (15.01)
25～	289 (21.57)	249 (21.80)	189 (14.00)	43 (18.22)	54 (17.31)	141 (19.83)	965 (18.96)
60～	55 (4.10)	44 (3.85)	21 (1.56)	13 (5.51)	3 (0.96)	9 (1.27)	145 (2.85)
**Occupation**							
Nursery children	401 (29.93)	328 (28.72)	357 (26.44)	60 (25.42)	28 (8.97)	157 (22.08)	1331 (26.14)
Scattered children	161 (12.01)	132 (11.56)	132 (9.78)	60 (25.42)	13 (4.17)	47 (6.61)	545 (10.71)
Students	353 (26.34)	327 (28.63)	552 (40.89)	39 (16.53)	187 (59.94)	317 (44.59)	1775 (34.87)
Cadre and employees	71 (5.30)	54 (4.73)	57 (4.22)	10 (4.24)	8 (2.56)	25 (3.52)	225 (4.42)
Workers	35 (2.61)	23 (2.01)	18 (1.33)	4 (1.69)	6 (1.92)	23 (3.23)	109 (2.14)
Farmers	83 (6.19)	53 (4.64)	61 (4.52)	15 (6.36)	3 (0.96)	23 (3.23)	238 (4.67)
Others	236 (17.61)	225 (19.70)	173 (12.81)	48 (20.34)	67 (21.47)	119 (16.74)	868 (17.05)
