# Supplementary figures and images for: Genomic Analyses Uncover Evolutionary Features of Influenza A/H3N2 Viruses in Yunnan Province, China, from 2017 to 2022
Source: Viruses. 2024 Jan 18;16(1):138. doi: 10.3390/v16010138 (PMC10820241; doi:10.3390/v16010138)

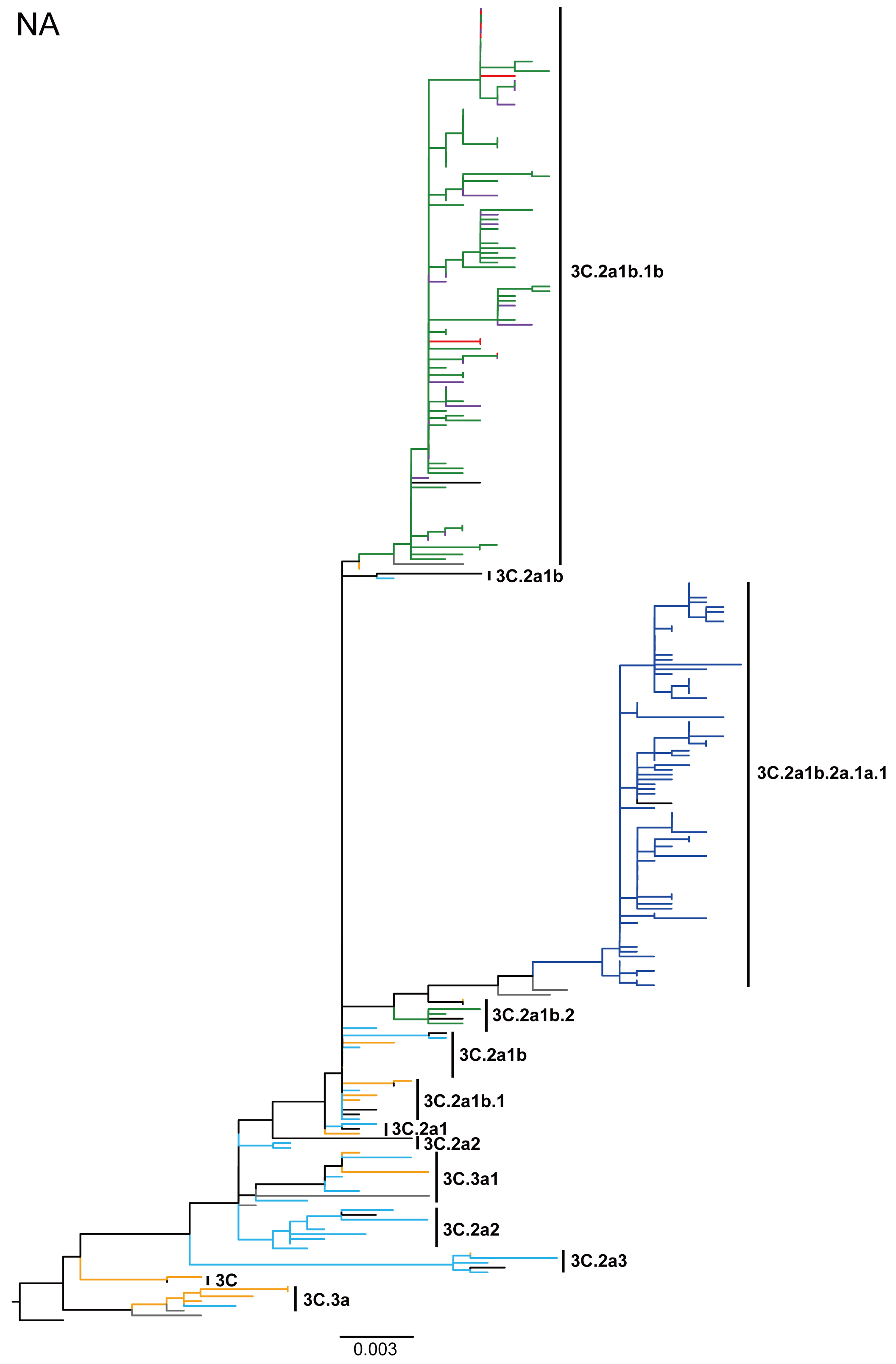

Supplement: Supplementary file 1 [file viruses-16-00138-s001.zip › Figure S1.tif]

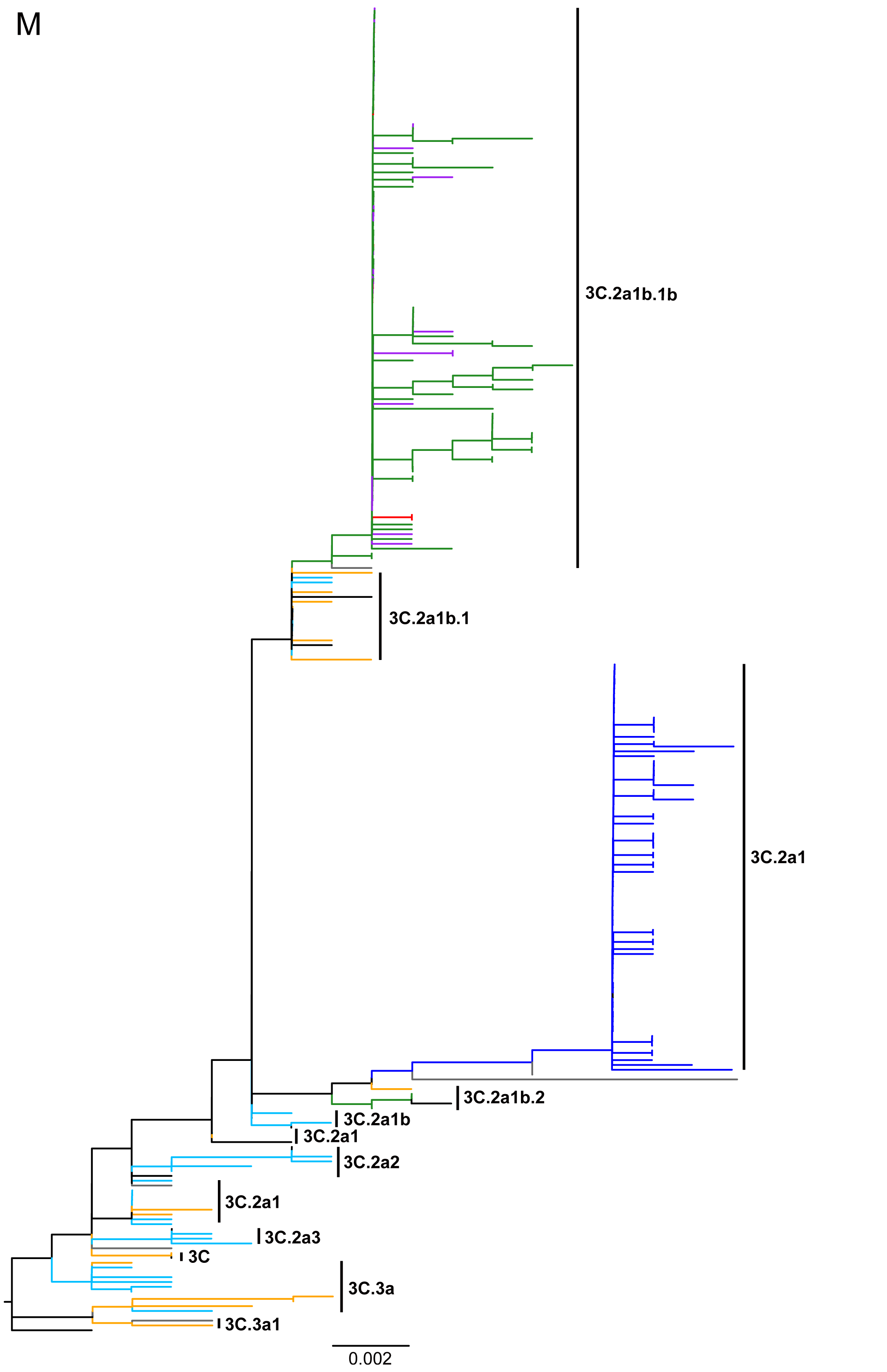

Supplement: Supplementary file 1 [file viruses-16-00138-s001.zip › Figure S2.tif]

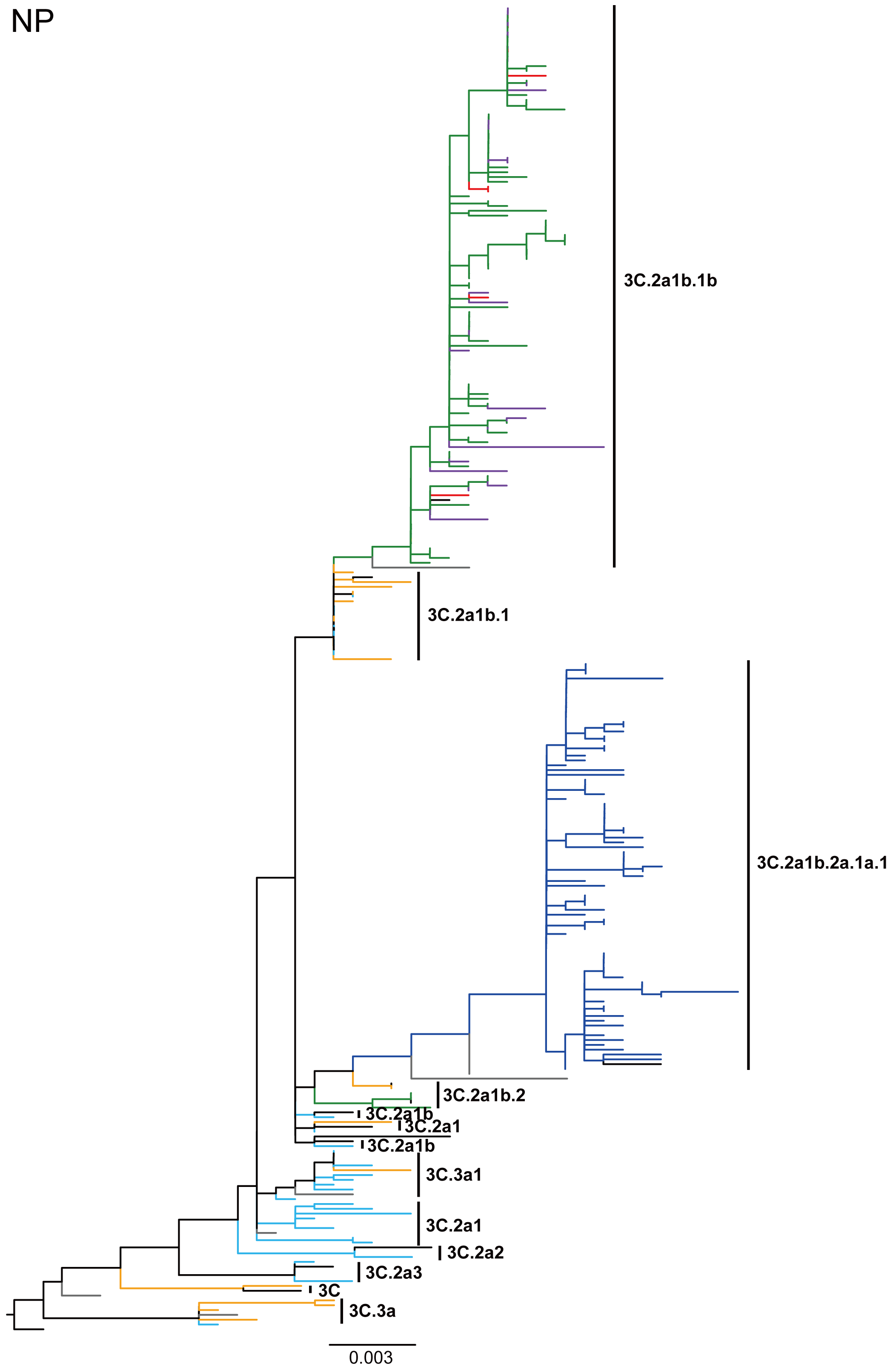

Supplement: Supplementary file 1 [file viruses-16-00138-s001.zip › Figure S3.tif]

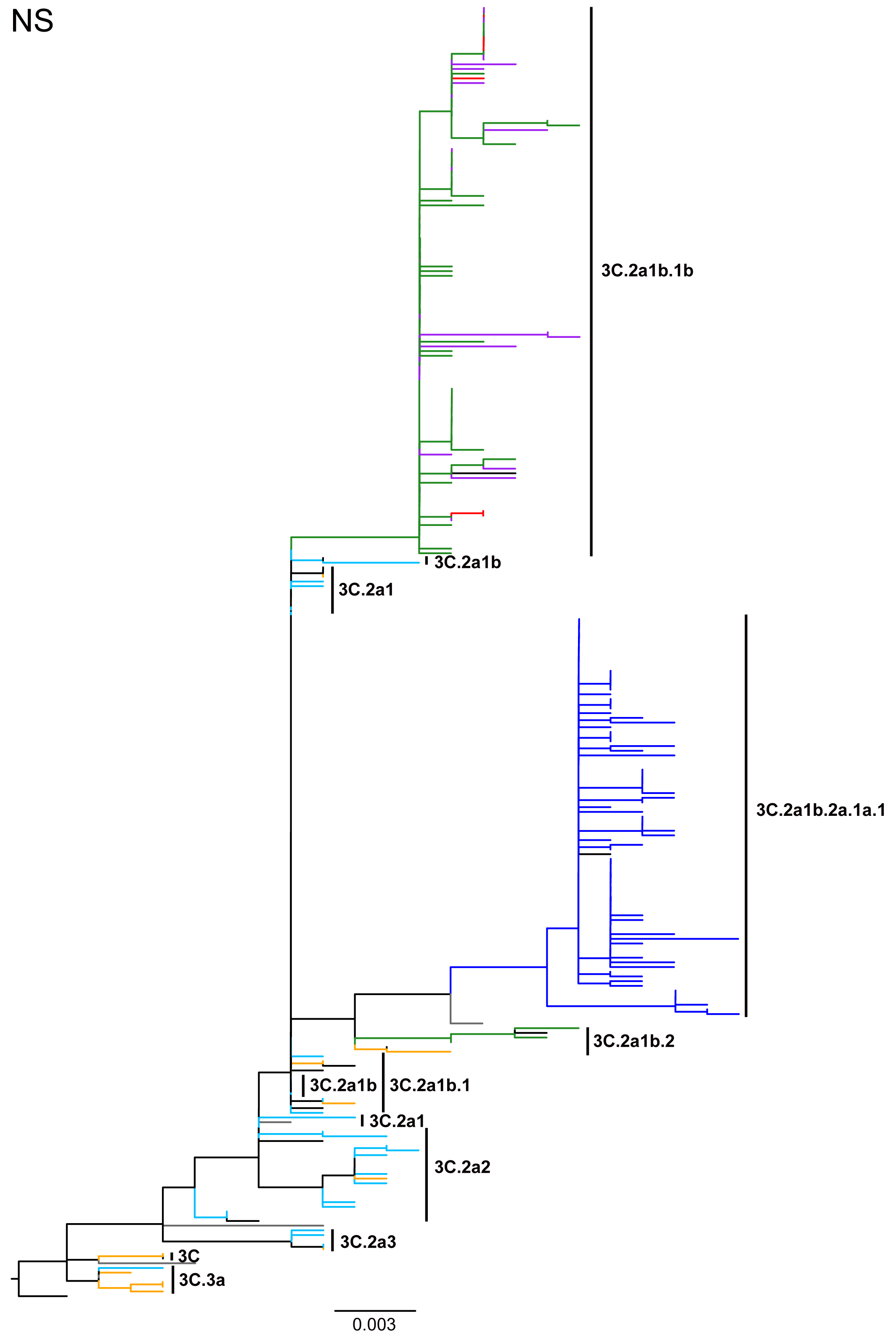

Supplement: Supplementary file 1 [file viruses-16-00138-s001.zip › Figure S4.tif]

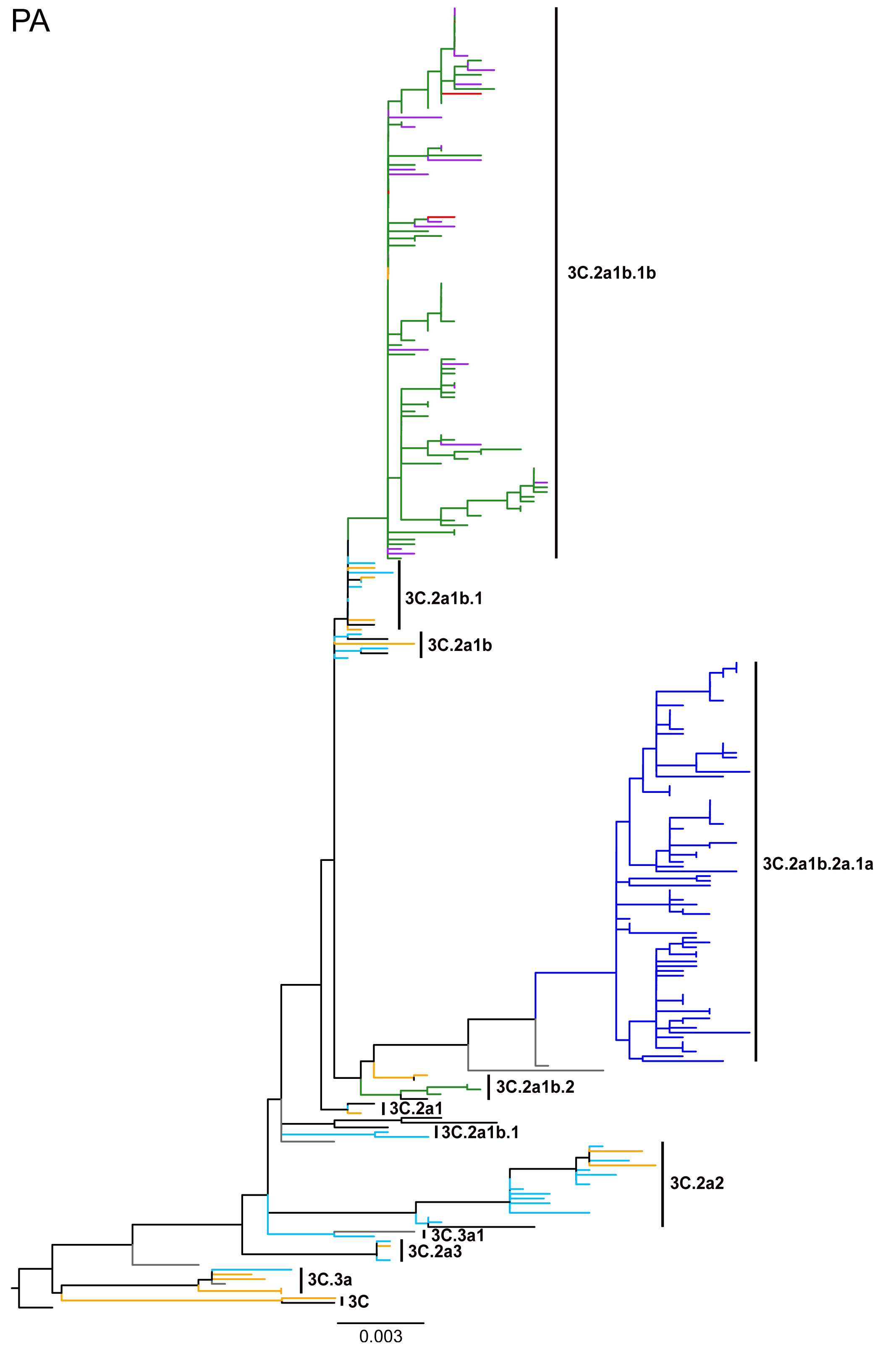

Supplement: Supplementary file 1 [file viruses-16-00138-s001.zip › Figure S5.tif]

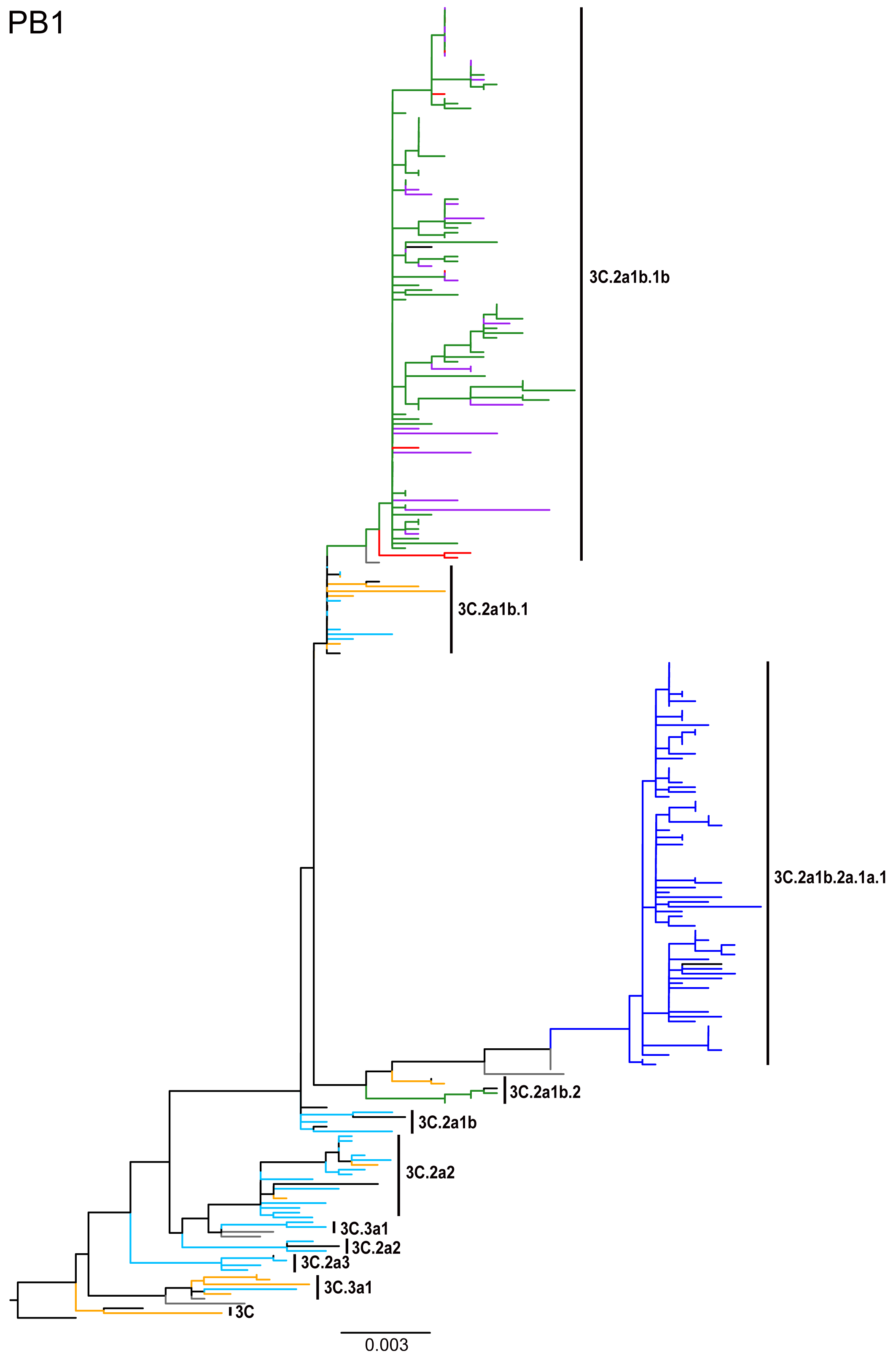

Supplement: Supplementary file 1 [file viruses-16-00138-s001.zip › Figure S6.tif]

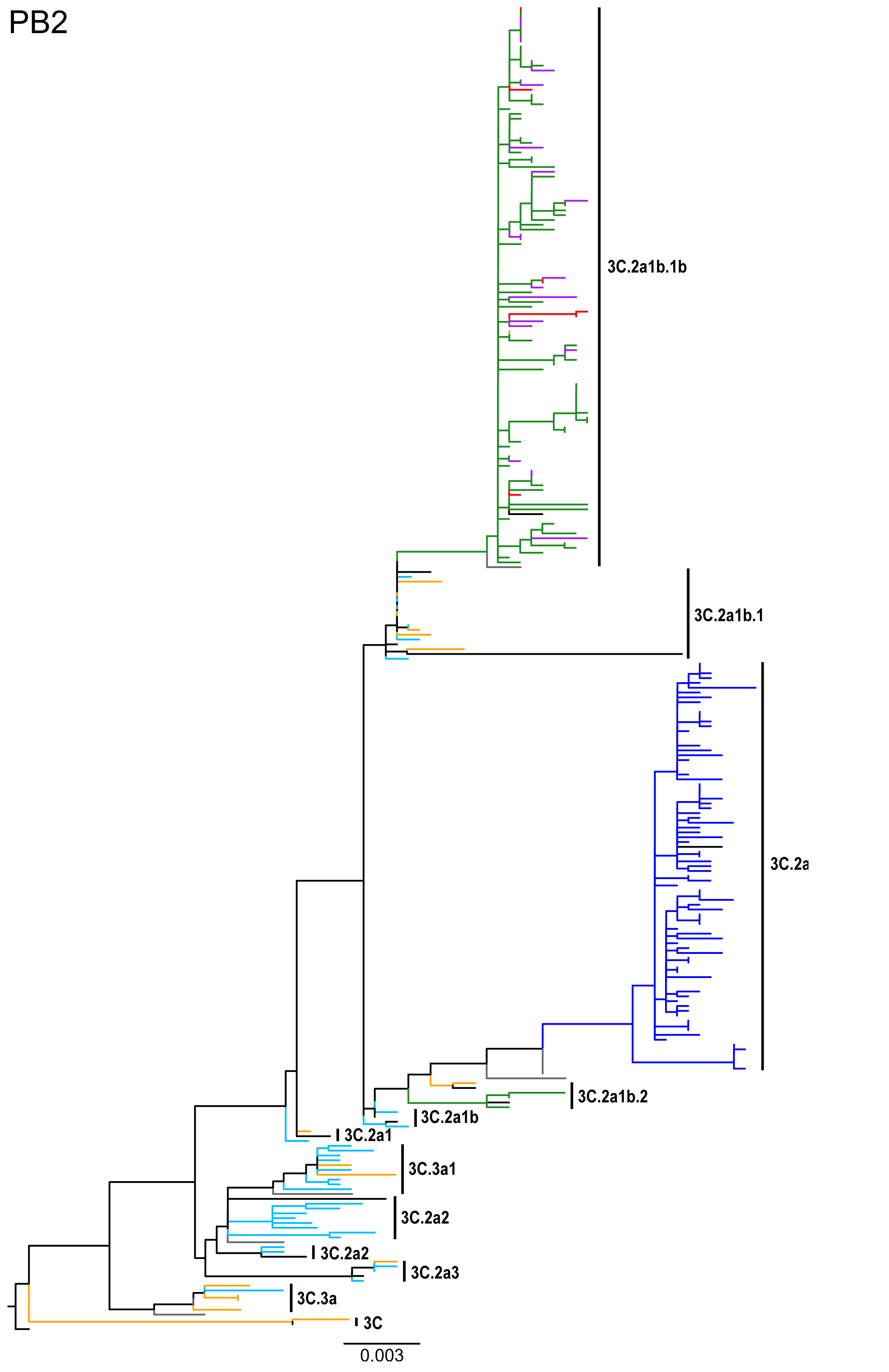

Supplement: Supplementary file 1 [file viruses-16-00138-s001.zip › Figure S7.tif]
